# Ginsenosides emerging as both bifunctional drugs and nanocarriers for enhanced antitumor therapies

**DOI:** 10.1186/s12951-021-01062-5

**Published:** 2021-10-15

**Authors:** Hong Wang, Yu Zheng, Qiang Sun, Zhen Zhang, Mengnan Zhao, Cheng Peng, Sanjun Shi

**Affiliations:** State Key Laboratory of Southwestern Chinese Medicine Resources, School of Pharmacy, Chengdu University of Traditional Chinese Medicine, Chengdu, 611137 China

**Keywords:** Ginsenosides, Antitumor, Delivery systems, Biomimetic, Bifunctional drug, Carrier, Unification of medicines and excipients

## Abstract

Ginsenosides, the main components isolated from *Panax ginseng*, can play a therapeutic role by inducing tumor cell apoptosis and reducing proliferation, invasion, metastasis; by enhancing immune regulation; and by reversing tumor cell multidrug resistance. However, clinical applications have been limited because of ginsenosides’ physical and chemical properties such as low solubility and poor stability, as well as their short half-life, easy elimination, degradation, and other pharmacokinetic properties in vivo. In recent years, developing a ginsenoside delivery system for bifunctional drugs or carriers has attracted much attention from researchers. To create a precise treatment strategy for cancer, a variety of nano delivery systems and preparation technologies based on ginsenosides have been conducted (e.g., polymer nanoparticles [NPs], liposomes, micelles, microemulsions, protein NPs, metals and inorganic NPs, biomimetic NPs). It is desirable to design a targeted delivery system to achieve antitumor efficacy that can not only cross various barriers but also can enhance immune regulation, eventually converting to a clinical application. Therefore, this review focused on the latest research about delivery systems encapsulated or modified with ginsenosides, and unification of medicines and excipients based on ginsenosides for improving drug bioavailability and targeting ability. In addition, challenges and new treatment methods were discussed to support the development of these new tumor therapeutic agents for use in clinical treatment.

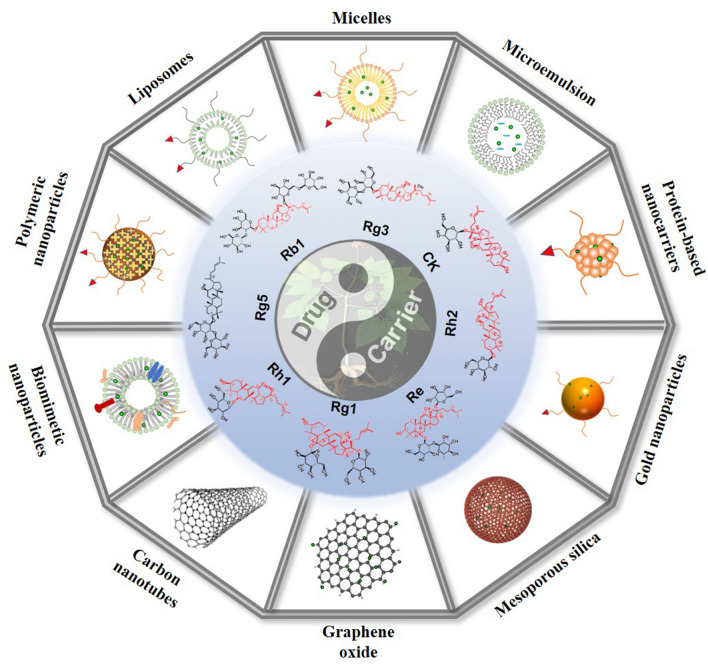

## Introduction

Drug delivery systems (DDSs) for cancer treatment, which have been explored for many years, have been developed rapidly for their solubility, bioavailability, and targeting with their high drugloading rates, large specific surface areas and diverse surface functions [1–3]. However, further work has been hindered by low drug-loading rates, drug resistance, toxicity and immune responses induced by nanocarriers [4, 5]. Because most DDSs acted only as excipients with no direct effects, short and long-term toxicity can appear with their metabolites.

In addition, therapeutic effects can be reduced for the phagocytosis and clearance of nanoparticles (NPs) by the reticuloendothelial system (RES). Furthermore, DDSs can interact with cell surface-specific receptors resulting in adverse immune reactions. Nevertheless, natural products such as ginsenosides have been studied widely for the treatment of cancer and other diseases because of their chemical and biological properties including chemical diversity, specificity, and low toxicity, making them conducive to the development of DDSs [6].

**Fig. 1:**
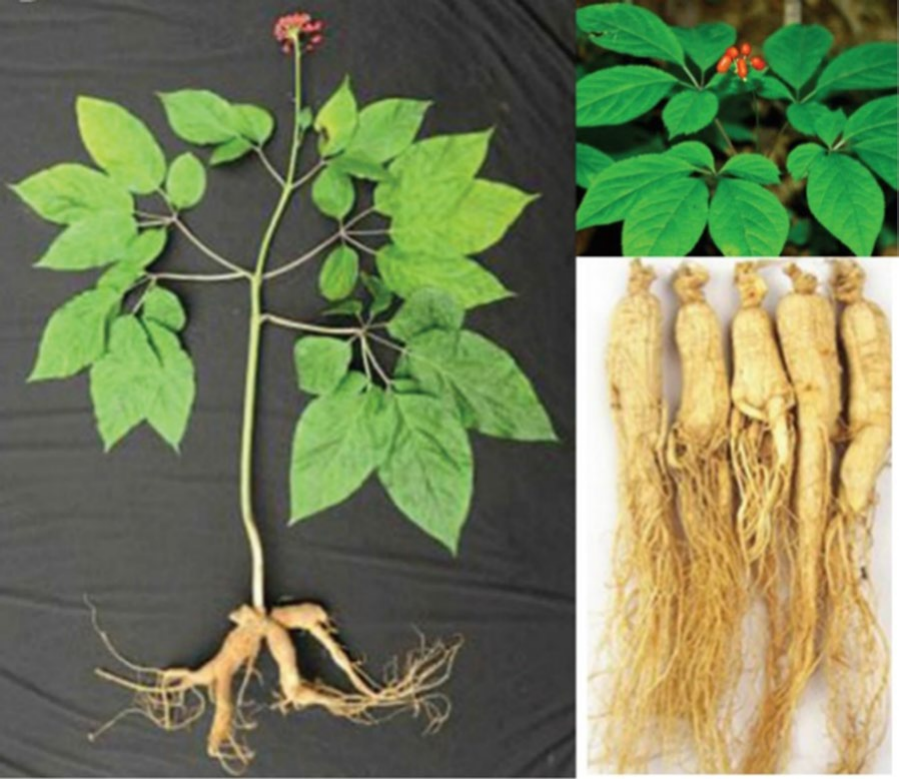
Source of ginsenosides. Reprinted with the permission from Ref [7]. Copyright © 2021 RSC

Ginsenosides are a group of bioactive compounds extracted from *Pan*a*x ginseng* [7] (Fig. 1). As small-molecule substances, ginsenosides can resist diabetes, depression and cancer, offering better protective effects for cerebral ischemia, endothelial cell injury, and cardiovascular disease (CVD) [8–11]. Currently, ginsenoside Rg3 has been launched as a new drug in traditional Chinese medicine (TCM, Shenyi capsule) for the treatment of lung, breast, gastrointestinal (GI) cancers [12]. Researches have reported that the human body can develop resistance after multiple administrations of cancer therapy. Ginsenosides combined with cisplatin [13], adriamycin [14], vincristine [15] or other chemotherapy drugs can reverse multidrug resistance and improve the antitumor effects for lung and liver cancers. Ginsenosides have been proved to exhibit good anticancer activity and targeting ability, both as a drug and as an excipient compound simultaneously [16–20]. However, due to their poor water solubility caused by the lipophilic steroid skeleton, GI instability [21], low oral absorption rate, short half-life, rapid clearance and other pharmacokinetic properties [22], most ginsenosides have a bioavailability of < 5% [23]. In addition, non-targeted aggregation can induce adverse reactions such as nerve, liver, and kidney toxicity, thus limiting clinical application [24]. Compared with others, more common drugs, ginsenosides’ solubility and absorption rate are improved by DDSs, with additional obvious targeting characteristics. Developing a new ginsenoside DDSs has attracted wide attention in attempts to achieve synergism and detoxification, as well as to improve bioavailability. Furthermore, because of the similar structure of cholesterol and ginsenosides, the latter have been used as excipients to synthesize liposomes with simultaneous targeting ability [25–28].

These findings have exploited the novel application of TCM, which is an important component of modern medical system. Researches on current, high-level, and novel preparations or dosage forms of TCM can provide new, high-quality therapy ideas. These ideas also can promote the TCM modernization process while protecting humans against diseases and poor health.

In this review, a variety of ginsenosides delivery systems and preparation technologies were examined, including polymer NPs, liposomes, micelles, microemulsions (MEs), protein NPs, metal and nonmetallic NPs, and biomimetic NPs. The permeability and retention (EPR) effect, as well as the recognition between ligands and receptors, were utilized to target tumor cells to increase the curative effect. Nanomedicines have shown a variety of advantages in the treatment of tumor diseases by providing controlled-release and targeted drugs. However, a lack of toxicity research has affected the evaluation of their safety. Therefore, on the basis of the above information, this review not only examined the various nano DDSs of ginsenosides but also analyzed their function in improving bioavailability and targeting, in reducing toxicity, and in enhancing immune regulation. In addition, this review discussed the challenges of integrating nanomaterials into diagnosis and treatment, of transforming clinical practice, and other related topics.

## Ginsenosides

### Properties

Different ginsenosides have similar chemical structures of tetracyclic triterpenoid saponins composed of aglycones and glycosides, which typically contain a dammarane skeleton with 17 carbon atoms in its 4 rings and sugar groups that bond to the C-3 or C-20 position [29]. In their hydrophilicity chemical structure, ginsenosides’ solubilities depend on the amount of sugar moieties with a positive correlation. However, most ginsenosides with anticancer activity have exhibited low water solubility due to the lack of sugar moieties.

Because of the large molecular weight of the tetracyclic triterpenoid saponins, ginsenosides have shown poor permeability. Ginsenosides, which are absorbed in the GI tract mainly through sodium glucose cotransporter 1, have shown a deficient amount of absorption. After oral administration. On the other hand, after oral administration, ginsenosides exhibited poor stability in the GI tract, owing to the easy hydrolysis and metabolism of C-3 and C-20 glycosyls by GI enzymes or bacteria [30]. In addition, the amount of the original drug entering the blood circulation decreased because Rg3 and other ginsenosides were metabolized in the intestinal mucosa and liver before being absorbed into the blood circulation after GI administration [23, 31]. Therefore, it is necessary to design an appropriate DDSs to improve the water solubility, stability and permeability.

### Classification

Most ginsenosides have a steroid-like structure with 4 rings and sugar moieties, that produce various pharmacologies and bioactivities due to their tiny variations. To date, ≥ 100 types of ginsenosides have been extracted and reported [29]. Each type has a different number and site of glycosyl units at C-3, C-6 or C-20 binding to the hydroxyl groups.

**Fig. 2:**
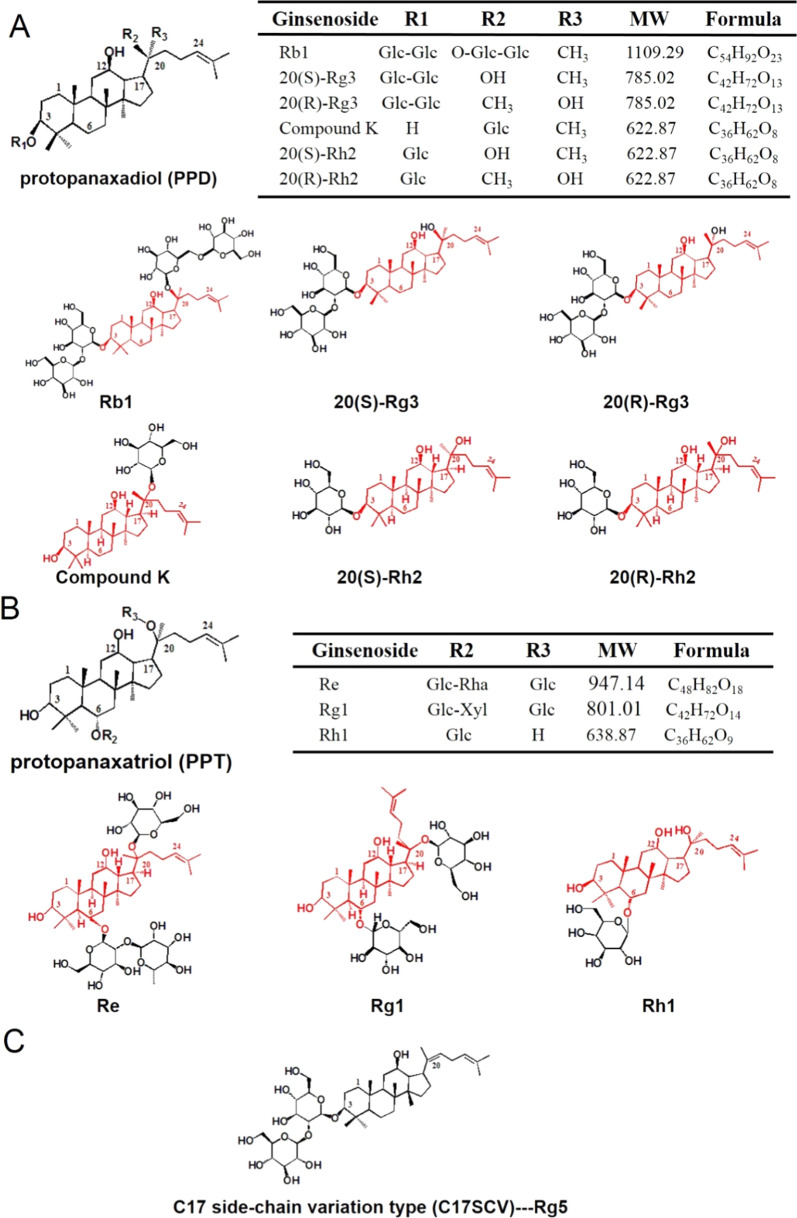
Chemical structures of ginsenosides extensively investigated in DDSs. **A** PPD type, **B** PPT type, **C** C17SCV type

Common types of ginsenosides are protopanaxadiol (PPD), protopanaxatriol (PPT), oleanolic acid and C17 side-chain variation type (C17SCV) sapogenins based on chemical structure [32–34]. Among them, the sugar moieties in the PPD group are mostly bound to C-3 of the dammarane-type triterpenoid saponins including ginsenosides Rb1, Rg3, Rh2, and compound K (CK). Moreover, the sugar moieties in the PPT group are bound mostly to C-6 of the dammarane-type triterpenoid saponins including ginsenoside Re, Rg1, and Rh1. Rg5 belongs to C17SCV (Fig. 2).

### Pharmacokinetics

Evaluating the pharmacokinetics and bioavailability of ginsenosides is beneficial for planning a dosage regimen and improving clinical efficacy. The pharmacokinetic evaluations of ginsenosides have been investigated in rats, with the ginsenosides following oral administration being poorly absorbed with low absolute bioavailability in vivo (Table 1). This low bioavailability has been ascribed to undesirable physicochemical properties such as poor solubility, low membrane permeability, poor GI stability, and easily metabolization in the intestinal mucosa and liver [11, 17, 35–39].

**Table 1:** The pharmacokinetics of ginsenosides evaluated in rats

Ginsenosides	Bioavailibility (%)	Dose (mg/kg)	Refs.
Rb1	4.35	50	[35]
Rg3	2.63	50	[36]
Compound K	35	20	[11]
Rh2	6.4	9	[17]
Re	7.06	10	[37]
Rg1	1.52–6.6	50	[38]
Rh1	1.01	5	[39]

### Functions

#### Pharmacological activities of ginsenosides as drugs

**Fig. 3:**
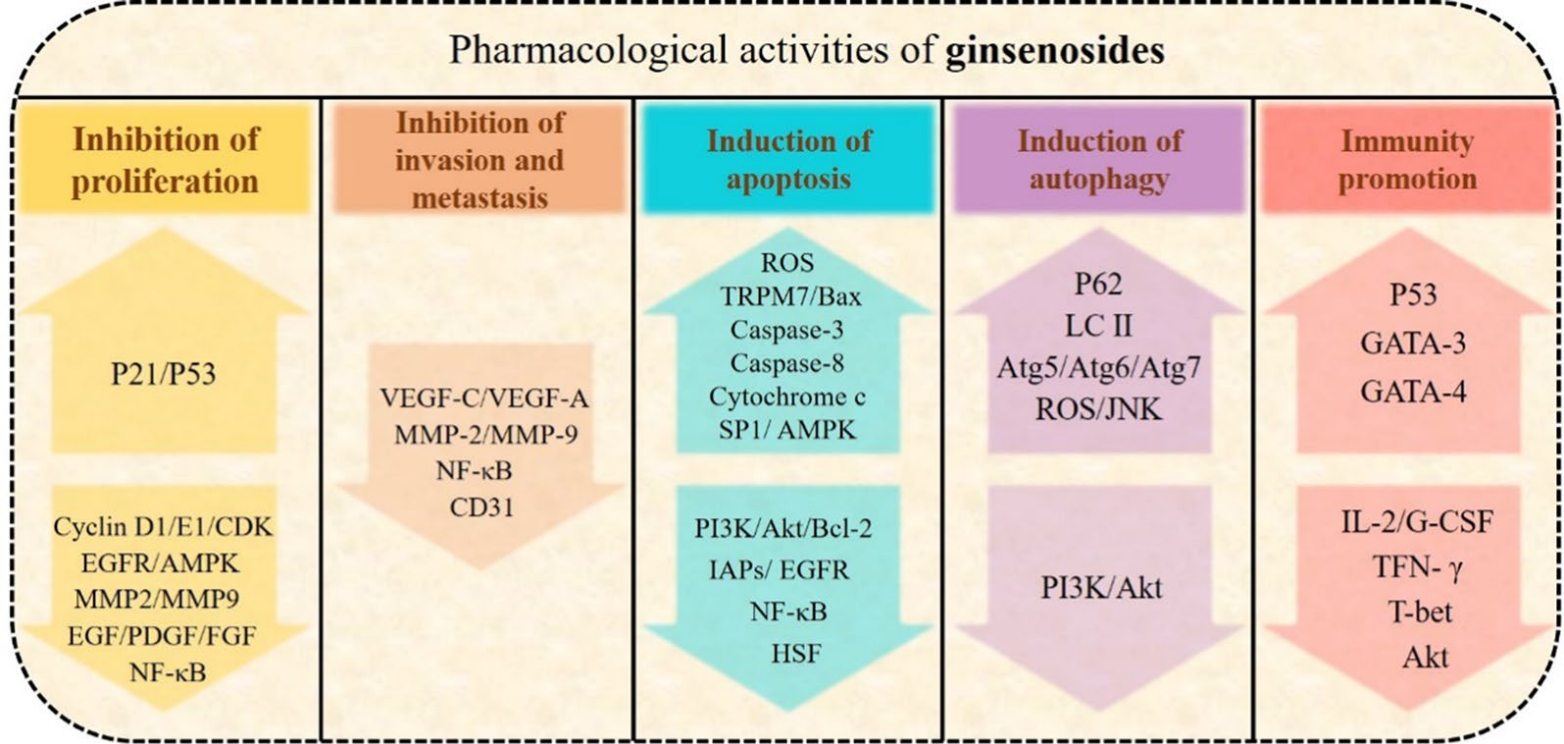
Anticancer activities of ginsenosides. The arrow upward in the figure indicates the upregulation of gene expression; the arrow downward indicates the downregulation of gene expression. *P21* cyclin-dependent kinase inhibitor, *P53* tumor suppressor and transcription factor, *CDKs* cyclin-dependent kinases, *EGFR* epidermal growth factor receptor, *AMPK* 5 AMP-activated protein kinase, *MMP* matrix metalloproteinase, *EGF* epithelial growth factor, *FDGF* platelet derived growth factor, *FGF* fibroblast growth factor, *NF-κB* nuclear factor κB, *VEGF* vascular endothelial growth factor, *CD31* Platelet endothelial cell adhesion molecule-1, *ROS* reactive oxygen species, *TRPM7* transient receptor potential melastatin 7, *Bax* bcl2 associated X protein, *SP1* transcription factor Sp1, *PI3K* phosphatidylinositol 3-kinase, *Akt* protein kinase B, *Bcl-2* B-cell lymphoma-2, *IAPs* inhibitor of apoptosis proteins, *EGFR* epidermal growth factor receptor, *HSF* the heat shock factor, *P62* sequestosome 1, *LC3-II* the processed form microtubule-associated protein 1 light chain 3, *Atg* autophagy-related protein, *JNK* c-Jun NH2-terminal kinase, *GATA* T cell specific transcription factor, *IL-2* Interleukin-2, *G-CSF* granulocyte colony-stimulating factor, *TNF* tumor necrosis factor, *T-bet* T-box transcription factor

Ginsenosides are one of the most common natural products, and their synthetic active ingredients have been used in medicine to prevent and treat various diseases, with a variety of pharmacological effects, including immune regulation and antiinflammatory and antitumor activities (Table 2) (Fig. 3) [40, 41]. Ginsenosides can resist diabetes, depression, and cancer, and also can exhibit better protective effects on cerebral ischemia, endothelial cell injury, and CVD [42–44]. In the clinic, ginsenosides combined with chemotherapy are frequently used to reduce the side effects of anticancer drugs such as cisplatin [45]. The anticancer activities of ginsenosides and their metabolites have complicated antitumor mechanisms; they have achieved antitumor effects mainly through inhibiting tumor cell proliferation, invasion, and metastasis; inducing tumor cell apoptosis, autophagy, and cell cycle arrest; and enhancing cell immune regulation [29].

**Table 2:** Anti-cancer activities of ginsenosides in several cancers

Cancer	Cell types	Ginsenosides	Outcomes	Mechanisms	Refs.
Gastrointestinal cancer	C26	Rb1	Amelioration of the inflammatory	Ameliorating expression of TNF-α and IL-6	[46]
	SW480, HT29, HCT116, Caco-2	Rg3	Inhibition of proliferation and growth, migration and invasion, induction of apoptosis	Inhibition of Wnt/ß-catenin/C/EBPβ/NF-κB signalling, decreasing the expressions of lncRNA CCAT1	[18, 47, 48]
	HCT116, HT-29	CK	Induction of autophagy and apoptosis	Activation of JNK and generation of ROX, activation of *caspase-9* and *caspase-3*, modulation of mitochondria-dependent and MAPK pathway	[49–51]
	HCT116, SW620,HCT-8, LoVo	Rh2	Induction of viability, proliferation and migration	Decreasing expressions of IL-6-induced signal transducer, STAT3, MMPs, MRP1, MDR1, LRP and GST	[52, 53]
	AGS	Re	Inhibition of proliferation, induction of apoptosis	Inducing S phase arrest via upregulating of p21, activation of *caspase-8, caspase-9,* and *caspase-3*	[54]
	SW620	Rh1	Inhibition of proliferation, migration and invasion	Activation of MAPK signaling, decreasing expressions of MMP1and MMP3, and increasing expressions of TIMP3	[55]
	BGC-823, AGS	Rg5	Inhibition of proliferation and migration	Inducing G_2_/M phase arrest, autophagy and apoptosis via regulating MAPK signalling	[56]
Breast cancer	MDA-MB-231, MDA-MB-453, BT-549	Rg3	Induction apoptosis	Inhibiting NF-κB signaling, regulating *Bax*/*Bcl-2* expression	[29, 57]
	MCF-7	Rh2	Inhibition of proliferation	Inducing G_1_-S phase arrest by knockdown of p15^Ink4B^ and p27^Kip1^	[58, 59]
	MCF-7	Rg5	Induction of apoptosis and autophagy	Inhibition of PI3K/Akt/mTOR pathway	[60, 61]
Lung cancer	NCI-H1650, H520, H1963	Rg3	Induction apoptosis	Inhibition of Notch/HES1 pathway	[57]
	A549	Rh2	Induction of proliferation and invasion	Inhibition of Wnt and hedgehog signaling	[62]
	HeLa, A549	Rg5	Induction of migration	Inhibited NF-κB signaling, attenuating expression of EphA2	[63]
Melanoma	B16-F10 melanoma	Rh2	Enhancing immune regulation	Enhanced CD4^+^ and CD8a^+^ T-lymphocytes infiltration	[64]

##### Inhibition of tumor proliferation

Ginsenosides have been proved to inhibit tumor proliferation. Ginsenoside Rg3 has shown the strongest effects [65], exhibiting excellent antiproliferation activity by inhibiting the expression of biomarker genes such as prostate specific antigen, 5 alpha reductase, and proliferating cell antigen [66]. In addition, ginsenosides can target the cell cycle arrest signaling pathway to inhibit cell growth. Cyclin D1, cdks 2/4/6 and other proteins are regulated by the *p21* gene, which are upregulated by Rh2 to induce arrest in the G_1_ phase of the cell cycle. Studies have illustrated that ginsenosides exerts an antiproliferation effect by increasing the expression of the *p53* gene in the G_2_ phase and releasing cytochrome c from mitochondria [67, 68].

##### Inhibition of tumor invasion and metastasis

Tumor invasion is closely related to the extracellular matrix and basement membrane proteolytic enzymes. Matrix metalloproteinases (MMPs), which depend on the metal ions, play an important role in the invasion and metastasis of tumor cells. Ginsenosides and their metabolites have displayed obvious inhibitory effects on both. Ginsenoside Rg3 and Rh2 can significantly inhibit the expression of MMP-2 and MMP-9 [69, 70].

##### Promoting tumor apoptosis

Apoptosis, the spontaneous and orderly death of cells controlled by genes, including death receptor, mitochondrial, and endoplasmic reticulum stress apoptosis, is accompanied by the activation of caspase. In colorectal cancer cells, the expression level of apoptosis gene *bcl-2* can be reduced by Rh2, while the expression of *caspase-3* is increased, eventually inducing apoptosis. In addition, mitochondria are the regulatory center of apoptosis, and mitochondrial apoptosis can be activated to regulate *bcl-2*, *bax*, *cytochrome c*, and reactive oxygen species (ROS) [71]. CK has been found to generate the disappearance of the mitochondrial membrane potential and activate the expression of *caspase-3* and *caspase-9* while releasing cytochrome c, thus inducing apoptosis [51].

##### Inducing tumor autophagy

Autophagy is the process of programmed cell death, in which cells controlled by autophagy-related genes combine with lysosomes to destroy damaged proteins and organelles. Ginsenoside Rg3 can induce autophagy in the HeLa cells by increasing the transformation of the microtubule associated protein light chain 3 [72]. In addition, ginsenoside CK can achieve autophagy by activating the AMPK/mTOR and JNK signals in the A549 cells [73].

##### Enhancing immune regulation

Immunotherapy, the treatment method of activating the immune system of the human body by medications, can produce many of the active immune cells to clear the tumor cells. Ginsenoside Rh2 has been shown to increase the number of T cells in mice with melanoma, through promoting the infiltration of CD4^+^ and CD8^+^ T cells to achieve an antitumor effect [64]. In addition, ginsenoside Rg3 has exhibited a strong immunomodulatory activity, which can maintain the balance of Th1/Th2 to enhance the immune function by regulating the cytokines and transcription factors [74].

#### Encapsulated activities of ginsenosides as carriers

Ginsenosides also can be utilized as carriers in the preparation of nanodrugs. Ginsenosides can stabilize the phospholipid bilayer for the amphiphilic structure. Because some ginsenosides share the same structure, a cholesterol-like steroidal mother nucleus, ginsenosides Rg3, Rg5, and Rh2 have been substituted with cholesterol to fabricate a nano structured lipid carrier, including liposomes with multifunctional pharmacological activities [19, 20, 26].

##### Minimizing side effects

Liposomes containing cholesterol have shown certain antitumor effect, but side-effects include hyperlipidemia, pulmonary hypertension, and other diseases caused by the excessive absorption of cholesterol by the human body [75, 76]. In addition, the high content of cholesterol in the tumor microenvironment (TME) is closely related to tumor growth. Ginsenoside-encapsulated liposomes, which are different from cholesterol, have been investigated extensively for their anticancer properties while minimizing side-effects [26].

##### Active targeting function

Ginsenoside liposomes are prone to accumulate in tumors due to their properties for recognizing the glucose transporter (GLUT) carrier on the tumor cell membrane; they have stronger toxicities and targeting abilities to the BGC-823 and HGC-27 cells [26, 27]. It has been reported that Rh2-, Rg3-, and Rg5-liposomes are mainly taken up through the GLUT1 and SGLT1, as well as the GLUT5 and the GLUT2 pathways, respectively [26]. Furthermore, the active targeting of Rg5-encapsulated liposomes is achieved mainly by the GLUT1 pathway [27].

##### Enhancing biomimetic function

NPs, which are easily adsorbed by the opsonin proteins such as immunoglobulin (Ig) and the complex proteins, have been recognized and cleared easily by mononuclear phagocytes. It has been demonstrated that the stealth effect of NPs modified with ginsenosides reduced the adsorption of opsonins on the surface of the liposomes. Ginsenosides Rh2, Rg3 and Rg5 have shown a stealth effect due to the increased adsorption of apolipoprotein E, which can retard the absorption of macrophages to liposomes [25].

##### Enhancing immune regulation

Ginsenoside Rh2 as carriers also can play a role in reconstructing the TME by transforming tumor-associated macrophage 2 (TAM2) into TAM1 to promote the role of T cells by inhibiting the activities of signal transducers and transcription activators [25].

## Ginsenosides as bifunctional drugs and nanocarriers in DDSs

### Ginsenosides as biofunctional drugs

**Fig. 4:**
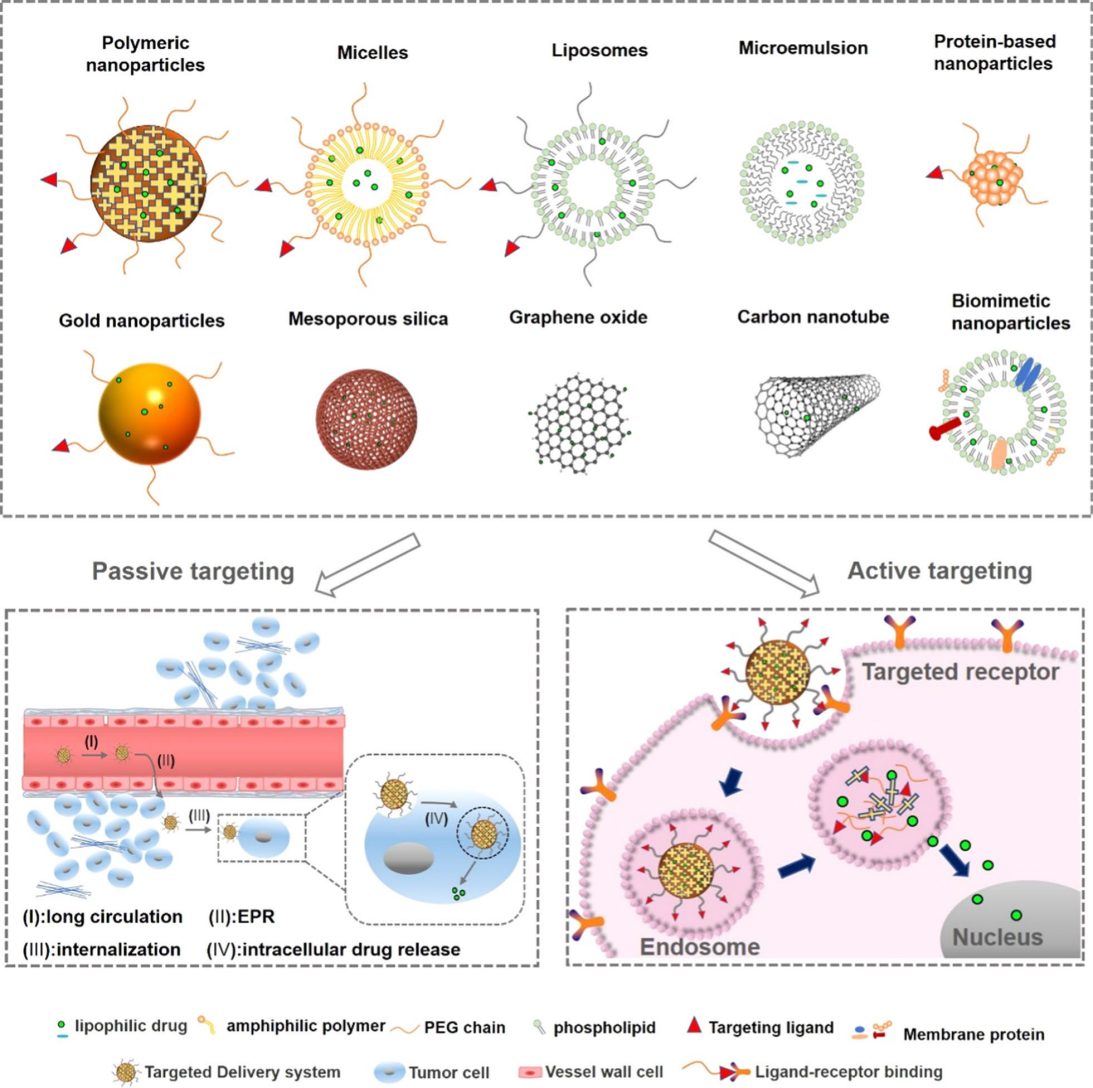
DDSs of ginsenosides and the passive and active targeting effects

In the application of nanomaterials in drug delivery, the selection of NPs is based on a drug’s physical and chemical properties. The combined application of nanoscience and bioactive natural compounds to create a safe, biodegradable, and biocompatible DDS has been studied often in recent years. Because ginsenosides have low aqueous solubility, membrane permeability, and bioavailability, as well as poor stability [77–79], these undesirable properties have limited their application in antitumor uses. A novel delivery system platform including polymeric NPs, liposomes, vesicular delivery systems, MEs, protein-based nanocarriers, metallic and inorganic NPs, and biomimetic NPs has been used to improve efficiencies and reduce the side-effects of ginsenosides (Fig. 4). Some nanocarriers have been utilized to lower the release rate of ginsenosides in vivo [80].

In addition, the novel carriers modified with specific moieties had stronger capabilities to provide better-targeted treatment outcomes for tumor cells. As observed in various studies, some carriers are better suited for increasing the bioavailability of ginsenosides, enhancing the efficiencies of tumor treatment, and reducing toxicity and side-effects [81]. The delivery systems for different types of ginsenosides have been explored, showing improved properties (Tables 3, 4).

**Table 3:** Ginsenosides delivery systems and improved properties

Bioactive compound	Delivery system	Improved properties	References
Rb1	MEs	Controlled and sustained drug release	[102]
	CNTs	Enhanced cytotoxicity to MCF-7 and PANC-1 cells	[103]
Rg3	Polymeric NPs	Longer circulation time; sustained drug release; passive target drug delivery; enhanced anti colorectal cancer activity	[88]
		Crossed BBB; promoted uptake efficiency of C6 glioma cells	[89]
		Sustained drug release; enhanced cytotoxicity to A549 cells	[90]
	Liposomes	Enhanced cytotoxicity to A549 and HepG-2 cells; inhibited growth rate of tumor-bearing mice	[97]
		Sustained drug release	[96]
		Longer circulation time; active target drug delivery; significant synergistic effect with PTX for antitumor activity	[26]
		Longer circulation time; active target drug delivery; promoted C6 glioma cells uptake efficiency and tumor penetration; biomimetic property; inhibited growth rate of brain tumor with PTX	[28]
	MEs	Controlled drug release	[104]
CK	Polymeric NPs	Sustained drug release; enhanced cytotoxicity to HepG2 cells	[105]
	Liposomes	Longer circulation time; active target drug delivery; proapoptotic effects to A549 cells; inhibited growth rate of tumor-bearing mice	[101]
	Micelles	Sustained drug release; passive tumor targeting; proapoptotic effects; inhibited tumor cell invasion, metastasis, and outflow of A549 and PC-9 cells; inhibited growth rate of tumor-bearing mice;	[106]
		Longer circulation time; sustained drug release; passive tumor targeting; proapoptotic effects to A549 cells; inhibited growth rate of tumor-bearing mice	[107]
		Longer circulation time; sustained drug release; active target drug delivery; enhanced cytotoxicity to HepG2 and Huh-7 cells	[108]
		Longer circulation time; active target drug delivery; proapoptotic effects to A549 cells; inhibited growth rate of tumor-bearing mice	[109]
	GNPs	Proapoptotic effects to A549, HT29, and AGS cells	[110]
	MSNPs	Enhanced anti-HepG2, -A549 and -HT-29 colon cancer activity	[111]
Rh2	Polymeric NPs	Passive tumor targeting; enhanced antilung cancer activity	[87]
		Enhanced cytotoxicity to MCF-7 cells	[21]
	Liposomes	Inhibited the tumor growth of A549 cells; antiproliferation and proapoptotic effects on xenografted tumors; safer than cisplatin group	[98]
		Longer circulation time; passive tumor targeting; inhibited growth rate of HepG2 tumor-bearing mice	[100]
		Longer circulation time; active target drug delivery; significant synergistic effect with PTX for antitumor activity;	[26]
		Longer circulation time; active target drug delivery; proapoptotic effects to 4T1 cells; inhibited growth rate of tumor-bearing mice	[25]
	Micelles	Longer circulation time; sustained drug release; inhibited growth rate of A549 tumor-bearing mice	[112]
		Longer circulation time; sustained drug release; promoted uptake efficiency of A549 cells; antiproliferation and proapoptotic effects	[113]
	MEs	Longer circulation time; crossed intestinal barrier; enhanced cytotoxicity and proapoptotic effects to A549 cells	[114]
	Protein-based nanocarriers	Longer circulation time; enhanced cytotoxicity to A549, HepG2, and HT29 cells	[115]
	GO	Enhanced cytotoxicity to OVCAR3, MDA-MB, and A375 cells	[116]
	MSNPs	Enhanced anti-HepG2, -A549 and -HT-29 colon cancer activity	[111]
Re	Polymeric NPs	Enhanced cytotoxicity to MCF-7 cells	[21]
	CDs	Enhanced cytotoxicity to MCF-7, HepG2, and A375 cells	[117]
Rg1	CNTs	Enhanced cytotoxicity to MCF-7 and PANC-1 cells	[103]
Rh1	Polymeric NPs	Passive tumor targeting; enhanced antilung cancer activity	[87]
Rg5	Liposomes	Longer circulation time; active target drug delivery; significant synergistic effects with PTX for antitumor activity	[26]
		Longer circulation time; active target drug delivery; biomimetic property; inhibited growth rate of HGC-27, A549, and MCF-7 in tumor-bearing mice with PTX	[27]
	Protein-based nanocarriers	Longer circulation time; sustained drug release; active target drug delivery; proapoptotic effects to A549 cells;	[118]

*CK* compound K, *MEs* microemulsions, *CNTs* carbon nanotubes, *NPs* nanoparticles, *GNPs* gold nanoparticles, *MSNPs* mesoporous silica nanoparticles, *GO* graphene oxide, *CDs* carbon dots, *BBB* blood–brain barrier, *PTX* paclitaxel

**Table 4:** DDSs for ginsenoside studied in preclinical cancer models

DDS	Carrier	Bioactive compound	Target form/molecular	Cancer model	Loading efficiency	Encapsulation efficiency	Main results	References
Polymeric NPs	PEG-COOH	Rh2/Rh1	Passive tumor targeting	A549 cells	NA	NA	In vitro*:* PEG-Rh1 conjugate showed stronger anticancer activity in human non-small cell lung cancer cell line	[87]
	NA	Rh2/Re	NA	MCF-7 cells	38%/32%	NA	In vitro*:* GS-Rh2 showed significant cytotoxicity to MCF-7 cancer cells	[21]
	DA-OCMC	CK	NA	HepG2 cells	10.65% ± 1.49%	42.65% ± 1.24%	In vitro*:* CK-NPs showed dose-dependent inhibitory effects on HepG2 cells with IC_50_ values of 23.33 and 16.58 μg/mL	[105]
	mPEG-b-P (Glu-co-Phe)	20 (S)-Rg3	Passive tumor targeting	Colorectal cancer/ mice	8.90%	82.40%	In vitro*:* Drug-loaded NPs possessed longer circulation time in blood In vivo*:* Proliferation of tumors can be significantly inhibited by Rg3-NPs through reducing expression of proliferating cell nuclear antigen and inducing apoptosis by increasing expression of caspase-3 in subcutaneous colon cancer model in mice	[88]
	ANG-Rg3-NP	Rg3	NA	C6 glioma cells	27.2% ± 1.4%	80.6% ± 3.0%	In vitro*:* ANG-Rg3-NPs inhibited the proliferation of C6 glioma cells in a concentration-dependent manner. Angioprep-2 functionalized NPs were easier to cross the BBB and accelerate uptake of NPs by cells	[89]
	CS/HA /HPC	20 (R)-Rg3	NA	A549 cells	15.87% ± 0.09%	100.8% ± 6.1%	In vitro*:* Proliferation of A549 cells can be inhibited effectively by microparticles	[90]
Liposomes	ePC	Rg3	NA	A549 cells/ HepG2 cells/mice	NA	82.47% ± 0.74%	In vitro*:* Cytotoxicity of A549 and HepG-2 cells could be enhanced by L-Rg3 In vivo*:* C_max_ and AUC of L-Rg3 were 1.19 × and 1.52 × higher than those of Rg3. Growth rate of BALB/c nude mice inoculated with A549 tumor cells was significantly inhibited by L-Rg3. Besides, Tumor growth can be inhibited by liposome by reducing MVD and enhancing angiogenesis inhibition	[97]
	DSPE-PEG2000	Rg3	NA	NA	7.44% ± 0.08%	85.24% ± 1.02%	In vitro*:* Rg3-PEGylated liposomes showed sustained release	[96]
	DSPE-PEG2000	Rh2	NA	A549 cells/mice	15.3%	88.2%	In vitro*:* IC_50_ values of A549 cells treated with DLT indicated that tumor growth could be inhibited by DLT In vivo*:* DLT showed stronger antiproliferation and apoptosis effects on xenografted tumors. DDS was safer than cisplatin in treatment of tumors	[98]
	mPEG-PLA	Rh2	Passive tumor targeting	HepG2/mice	NA	94.93% ± 4.18%	In vivo*:* Fluorescence intensity at tumor site decreased gradually after injection of PLP for 8 h and lasted for 24 h. Rh2-PLP was superior to Rh2-LP and Rh2-CLP in anti-tumor effect	[100]
	EYPC/Rh2 /Rg3/Rg5	PTX/Rh2/Rg3 /Rg5	Active targeting: Rh2/Rg3/Rg5	BGC-823 cells/mice	Rh2: 5.6% ± 0.3% Rg3: 7.3% ± 0.4% Rg5: 4% ± 0.1%	Rh2: 91.3% ± 2.1% Rg3: 95.5% ± 3.3% Rg5: 82.8% ± 1.6%	In vitro*:* Ginsenoside liposome can be accumulated in tumor for recognizing GLUT carrier on tumor cell membrane In vivo*:* Ginsenosides showed significant synergistic effects with PTX for antitumor activity	[26]
	EYPC/Rh2	PTX/Rh2	Active targeting: Rh2	4T1 cells /mice	5.6%	91.3%	In vitro*:* PTX-Rh2-liposome showed ~ 80% cell apoptosis to 4T1 cells In vivo*:* PTX-Rh2-liposome reduced tumor growth to certain extent comparable with lipisu	[25]
	EPC/Rg3	PTX/Rg3	Active targeting: Rg3	C6 murine glioma cells/mice	9.80% ± 0.13%	94.15% ± 1.34%	In vitro*:* Rg3-liposome promoted C6 glioma cell’s uptake efficiency and tumor penetration simultaneously In vivo*:* PTX-Rg3-liposome showed antiproliferation effects. Immune microenvironment in glioma was activated, with promoting T cell immune response	[28]
	Lecithin/Rg5	PTX/Rg5	Active targeting: Rg5	HGC-27 /MCF-7 /A549 cells	NA	97.20%	In vivo*:* G-PTX achieved curative effects through targeting GLUT receptor on tumor surface In vivo*:* Broad-spectrum targeting ability of G-PTX was confirmed with HGC-27, A549, and MCF-7 subcutaneous tumor models, through clathrin and caveolae-dependent pathways for endocytosis	[27]
	DSPE-PEG2000-tLyp-1	CK	Active targeting: tLyp-1 peptide	A549 cells/mice	14.80%	83.40%	In vitro*:* tLyp-1 liposomes induced mitochondrial apoptosis of A549 tumor cells against tumor In vivo*:* tLyp-1 liposomes showed stronger antitumor effect and fewer side-effects on normal tissues than drug combinations	[101]
Micelles	pNP-PEG-pNP	Rh2	NA	A549 cells	NA	85.23% ± 4.38%	In vitro*:* CG-M showed stronger cell uptake ability, apoptosis induction ability and antiproliferation activity of A549 cells	[113]
	Solutol HS15/TPGS	Rh2	NA	A549 cells	7.68% ± 1.34%	95.27% ± 1.26%	In vitro*:* Rh2-M synthesized with Solutol HS15 and TPGS were capable of enhancing solubility and antitumor effects of Rh2 In vivo*:* Rh2-M displayed a higher tumor inhibition rate in tumor-bearing nude mice	[112]
	TPGS/PEG-PCL	CK	Passive tumor targeting	A549 and PC-9 cells/ mice	11.19% ± 0.87%	94.60% ± 1.45%	In vitro*:* Growth of A549 and PC-9 cells could be inhibited by CK-M by blocking G_1_ phase. *Bax* and *Bcl-2* were regulated to promote tumor cell apoptosis and inhibit tumor cell invasion, metastasis, and outflow In vivo*:* CK-M micelles showed higher tumor inhibition and longer maintenance time of micelles in tumor tissue	[106]
	PC/DP	CK	Passive tumor targeting	A549 cells/ mice	11.76% ± 1.32%	NA	In vitro*:* Micelles exerted proapoptotic effects and antitumor efficacy against human lung carcinoma A549 cells In vivo*:* Micelles exhibited higher tumor inhibition than free CK through increased permeability and retention effects	[107]
	DA-OCMC /A54 peptide	CK	Active targeting: peptide A54	HepG2/Huh-7 cells	3.18% ± 1.49%	76.56%	In vitro*:* Cytotoxicity of APD-CK micelles to HepG2 and Huh-7 cells was significantly higher than that of free CK. APD-CK micelles could promote protein expression of *caspase-3, caspase-9,* and poly (ADP-ribose) polymerase	[108]
	AP/TPGS	CK	Passive tumor targeting	A549 cells/mice	13.26% ± 1.89%	91.34% ± 5.24%	In vitro*:* Mixed micelles induced cell apoptosis and inhibited cell migration by inducing cell cycle arrest in the G_0_/G_1_ phase of A549 cells In vivo*:* A549 lung cancer xenografts in mice showed that mixed micelles were an efficient tumor-targeting DDS with obvious antitumor effects	[109]
MEs	PLA	20 (R)-Rg3	NA	NA	0.2853	0.78	In vitro*:* Ginsenoside Rg3 PLA microspheres exhibited controlled release of drugs	[104]
	PLGA	ac-Rb1	NA	NA	NA	0.96	In vitro*:* Controlled release of ac-Rb1 followed the Fickian diffusion	[102]
	Etoposide, coix seed oil	Rh2	NA	A549 cells/mice	NA	0.9	In vitro*:* Cytotoxicity and apoptosis induced by ECG-MEs were significantly enhanced in A549 cells In vivo*:* Oral ECG-MEs could enter blood circulation through intestinal barrier, then prolonged blood circulation time and accumulated in tumor site. Mechanism of antitumor effect was related to small-scale mediated tumor penetration depth and increased serum Th1 cytokine concentration	[122]
Protein-based nanocarriers	BSA	Rg5	Active targeting: FA	A549 cells/mice	12.64% ± 4.02%	73.59% ± 5.50%	In vitro*:* EPR effect and receptor-mediated targeting led to MCF-7 cell apoptosis In vivo*:* FA-modified targeted NPs efficiently accumulated Rg5 within 8 h at tumor site in MCF-7 xenograft mouse model, showing strong tumor aggregation capacity	[118]
	BSA	Rh2	NA	A549/HT29 cells	0.36 mg of Rh2/mg of BSA-Rh2 NPs	NA	In vitro*:* BSA-CK NPs had stronger inhibitory effects on lung cancer A549, HepG2 hepatoma, and HT29 colon cancer cell lines	[115]
GNPs	DCY51^T^-AuCKNps	CK	NA	A549/HT29 cells	11.03%	NA	In vitro*:* DCY51T-AuCKNp showed enhanced cell apoptosis in A549, HT29, and AGS cells, suggesting that DCY51T-AuCKNp was an effective photothermal agent with synergistic chemotherapy effects	[110]
Carbon nanomaterials	CDs	Re	NA	MCF-7/HepG2/A375	NA	NA	In vitro*:* Small-sized Re-CDs were beneficial to cellular uptake, which had strong fluorescence imaging properties. Re-CDs could inhibit tumor cell proliferation through ROS-mediated pathway	[117]
	CNTs	Rb1/Rg1	NA	MCF-7/PANC-1 cells	NA	NA	In vitro*:* Induction effect on MCF-7 and PANC-1 cell death pathway of ginsenoside CNT was stronger than pure ginsenoside	[103]
	GO	Rh2	NA	OVCAR3/MDA-MB/A375 cells	NA	NA	In vitro*:* Rh2, amino acid Lys and Arg modified GO showed higher antitumor activity and lowest toxicity to coagulation system and heart tissue	[116]
MSNPs	MSNPs	CK/Rh2	NA	A549/HepG2/HT-29 cells	NA	NA	In vitro*:* MSNPs enhanced efficacy of CK and Rh2, exerting anticancer effects on HepG2, A549 and HT-29 colon cancer cells	[111]

*DDS* drug delivery system, *NPs* nanoparticles, *MEs* microemulsions, *GNPs* gold nanoparticles, *MSNPs* mesoporous silica nanoparticles, *CK* compound K, *GO* graphene oxide, *CDs* carbon dots, *pNP-PEG-pNP* bis (4-nitrophenylcarbonate) polyethylene glycol, *TPGS/PEG-PCL* d-alpha Tocopheryl polyethylene glycol 1000 succinate/Poly (ethylene glycol)-poly (ε-caprolactone), *PC/DP* phosphatidylcholine/1,2-distearoyl-sn-glycero-3-phosphoethanolamine polyethylene glycol 2000, *DA-OCMC* deoxycholic acid-O carboxymethyl chitosan, *A54 peptide* liver cancer-specific binding peptide A54, *AP/TPGS* ascorbyl palmitate/d-α-tocopheryl polyethylene glycol 1000 succinate monoester, mPEG-b-P (Glu-co-Phe) poly (ethylene glycol)-block-poly (L-glutamic acid-co-l-phenylalanine), *ANG-Rg3-NP* angioep-2 polypeptide-Rg3, *CS* chitosan, HA hyaluronic acid, *HPC* hydroxypropyl cellulose, *ePC* yolk phosphatidylcholine, *DSPE-PEG2000* 1,2-distearoyl-sn-glycero-3-phosphoethanolamine poly (ethylene glycol) 2000, *EYPC* egg yolk lecithin, mPEG-PLA methoxy poly (ethylene glycol)-poly (lactide), *PTX* paclitaxel, *tLyp-1* peptide CGNKRTR, *PLA* polylactide, PLGA poly (dl-lactide-co-glycolide), *ac-Rb1* 6″-O-Acetylginsenoside Rb1, *BSA* bovine serum albumin, *FA* folic acid, *CDs* carbon dots, *CNTs* carbon nanotubes, *GO* graphene oxide, *DCY51*^*T*^ lactobacillus kimchicus, *MSNPs* mesoporous silica nanoparticles, *NA* not applicable, *BBB* blood–brain barrier, *AUC* area under the curve

#### Polymeric nanoparticles

Polymeric NPs are synthesized by self-assembly of amphiphilic surfactants in an aqueous phase, which can load ginsenosides into a core or onto the shell of the particles, with diameters usually ranging from 10 to 1000 nm [82–84]. Hydrophilic carriers such as polyethylene glycol (PEG), chitosan (CS), amphiphilic block copolymers, and polypeptides have been loaded on the surface of NPs. The polymeric nanoparticles not only exhibited good aqueous solubility, but also prolonged the release of ginsenosides in the physiological environment, which is useful for sustained-drug release and enhancing drug penetration and retention in tumors.

PEG is used as a hydrophilic carrier on the surface of NPs because of the small size of NPs and quick clearance by the kidneys [85]. Some researchers have pointed out that phagocytosis can be shielded not only by increasing a drug’s solubility in the blood, prolonging a drug’s blood circulation time, and by enhancing a drug’s penetration and retention in tumors but also by reducing the absorption of nonspecific proteins [86].

Ramya et al*.* [87] have prepared the effective spherical copolymers PEG-Rh1 and PEG-Rh2, with particle sizes of 62 ± 5.72 nm and 134 ± 8.75 nm, respectively. Furthermore, an effective ginsenosides DDS was fabricated with an aerosol solvent extraction system using ginsenosides Re and Rh2 as drug models. Remarkably, nanocomposites (NanoGS) exhibited a higher dissolution rate and significant cytotoxicity to the MCF-7 cancer cells than did the ginsenosides in vitro [21]. It has been verified that ginsenoside Rg3 modified with amino acids, peptides and CS can pass through the blood brain barrier (BBB) and prolong the blood circulation time of drugs in vivo. In addition, Qiu et al*.* have explored 20 (S)-ginsenoside loaded poly (ethylene glycol)-block-poly (l-glutamic acid-co-l-phenylalanine) (mPEG-b-P (Glu-co-Phe)) NPs, which can target cancer cells owing to pH sensitivity, finding a longer circulation time in the blood [88].

**Fig. 5:**
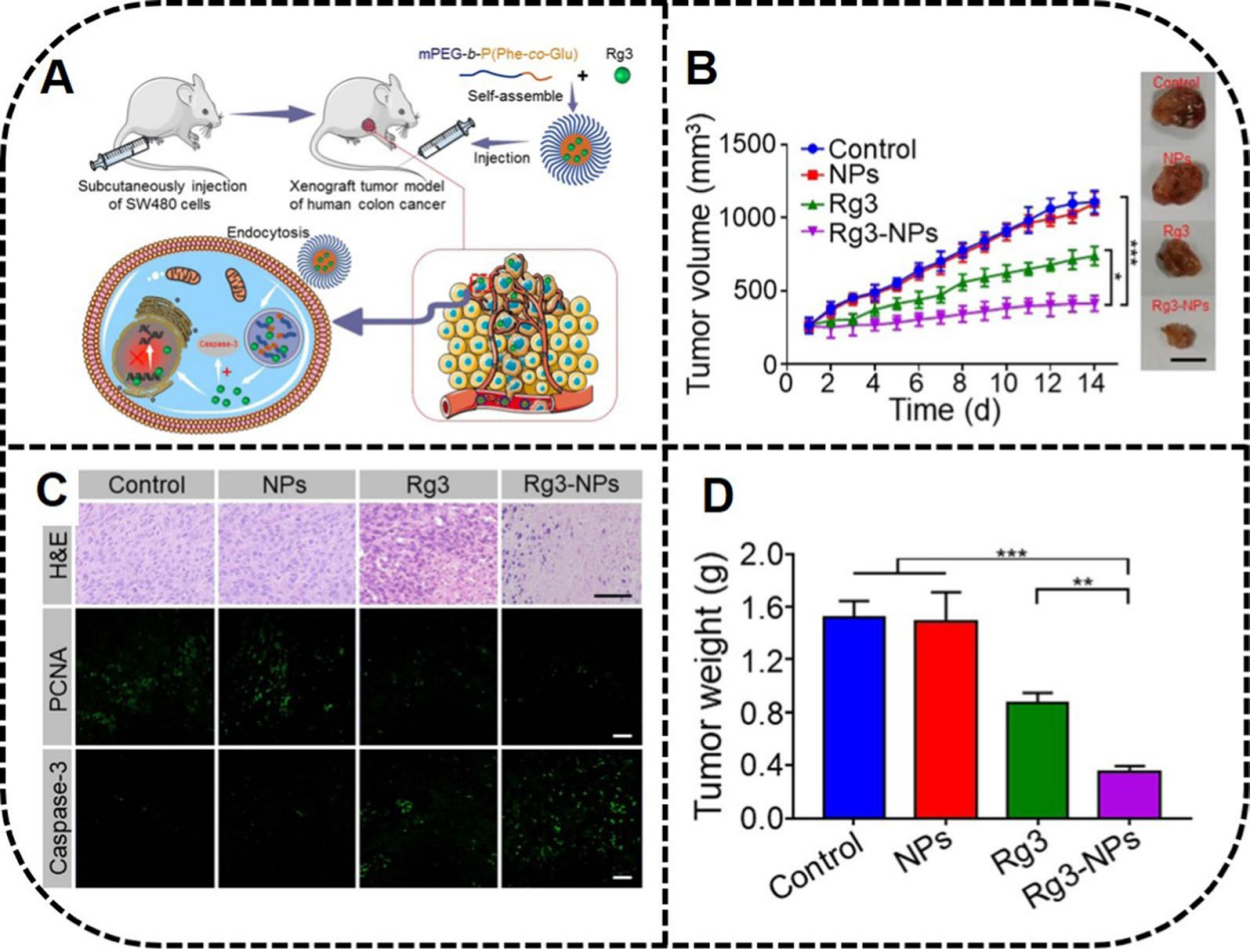
In-vivo anti-tumor activity by mPEG-b-P (Glu-co-Phe) Rg3 NPs. **A** Fabrication of mPEG-b-P (Glu-co-Phe) Rg3 NPs and their mechanism of preventing colorectal cancer by increasing the expression of *caspase-3*. **B** Tumor volume and images of mice treated with NPs. **C** H&E staining and immunofluorescence images of tumor tissues treated with NPs. **D** Tumor weight of mice in different groups. Reprinted with the permission from Ref [88]. Copyright © 2019 Springer

Furthermore, the expression of *caspase-3* in a subcutaneous colon cancer mouse model was increased by Rg3 NPs, which showed significant potential in the treatment of colorectal cancer (Fig. 5). Vascular endothelial cell-2 (angioep-2) polypeptides (ANG) also have been utilized to prepare NPs to load therapeutic macromolecules Rg3 [89]. Interestingly, ANG-Rg3-NP with a particle size of 147.1 ± 2.7 nm showed good sustained-release behavior, which inhibited the proliferation of C6 glioma cells in a concentration-dependent manner, while angioprep-2-functionalized NPs were easier to cross the BBB and accelerated the uptake of NPs by cells. As previously mentioned [90], using Rg3-loaded microparticles conducted by high-pressure homogenization combined with a spray drying method has shown that the expanded microparticles were prone to effectively inhibit the proliferation of A549 cells with good phagocytic function.

#### Liposomes

The lack of specificity, side-effects, and low solubility have limited the efficacy of ginsenosides. The further clinical application of ginsenosides in cancer treatment also has been hampered by poor bioavailability and rapid plasma elimination. However, liposome-based codelivery systems can increase drug solubility. In particular, liposomes are capable of delivering drugs to tumor cells to achieve a synergistic anticancer effect, which can be utilized to overcome the drug resistance of cancer cells [91, 92].

Liposomes typically are spherical colloidal particles similar to cell membranes with a lipid bilayer structure containing one or more amphiphilic bilayer membranes and internal water space. Liposomes have various merits in drug delivery, such as improving drug solubility, reducing side-effects, increasing free drug concentration in the vascular system, prolonging circulation time and targeting drug delivery [93–95]. Liposomes are classic and safe nanoscale formulation that can be transformed into effective clinical cancer treatment.

It has been demonstrated that PEGylated liposomes loading ginsenoside Rg3 display sustained-release and enhanced therapeutic effects with longer blood circulation time and enhanced drug uptake [96, 97]. In particular, the growth rate of BALB/c nude mice inoculated with A549 tumor cells was inhibited significantly by L-Rg3 after intravenous injection of the drug, which subdued the tumor growth through reducing microvessel density (MVD) and enhancing angiogenesis inhibition [97]. In addition, Jin et al*.* have proposed a multidrug-loaded system (CLT) combining betulinic acid, parthenolide, honokiol and ginsenoside Rh2 in a liposome system, demonstrating a safer DDS as compared to the cisplatin group in tumor treatment [98].

**Fig. 6:**
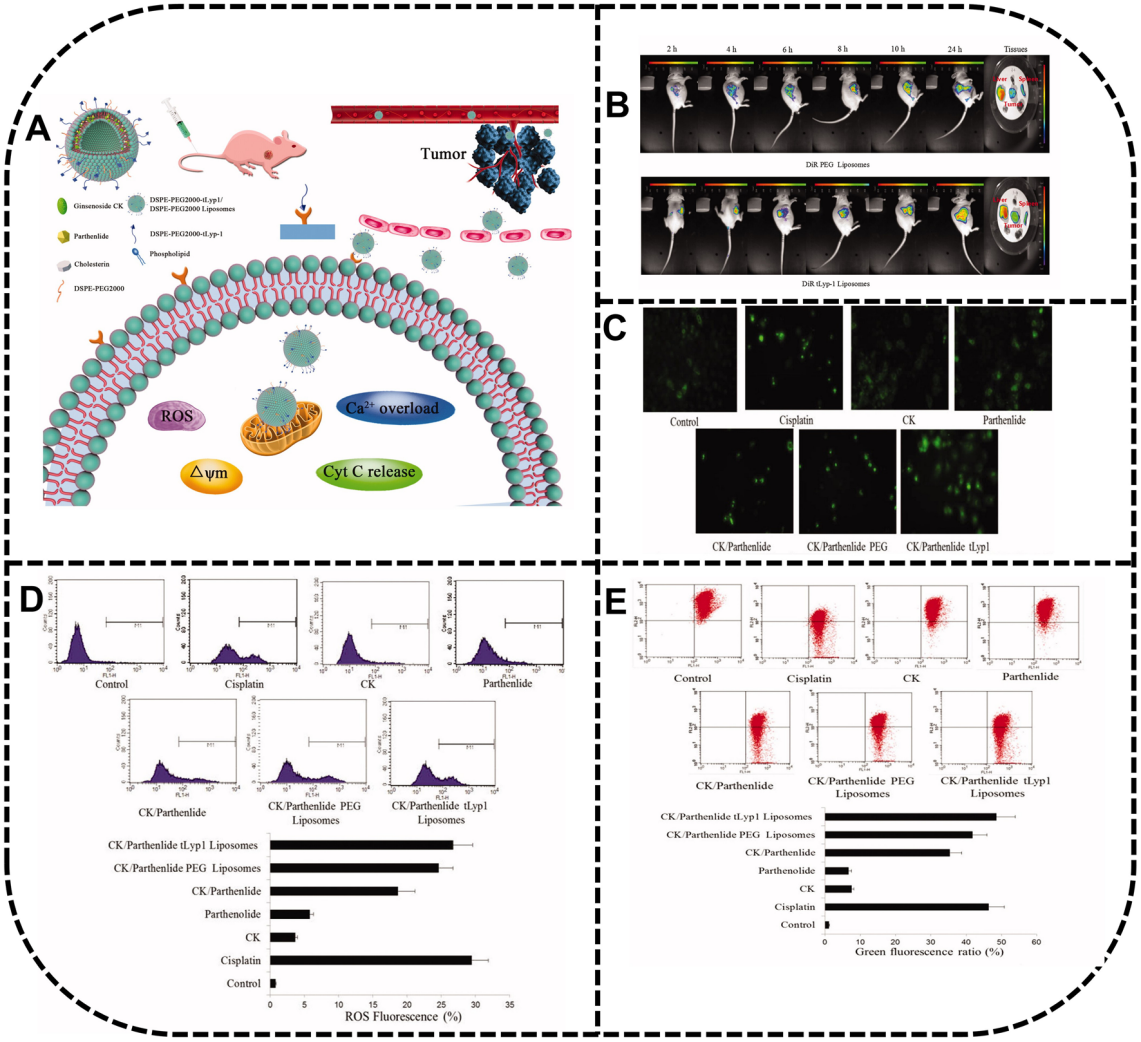
In-vivo antitumor activity by CK/tLyp-1 liposomes. **A** The mechanism of antitumor effect by CK/tLyp-1 liposomes. **B** The active targeting of CK/tLyp-1 liposomes in vivo. **C** An increased Ca^2+^ level of A549 cell treated with CK/tLyp-1 liposomes. **D** An increased ROS level of A549 cells treated with CK/tLyp-1 liposomes for 72 h. **E** Effects on MMP observed in A549 cells. Reprinted with the permission from Ref [101]. Copyright © 2018 Taylor & Francis

Most traditional liposomes lack resistance to RES clearance and selectivity to tumor sites, which can result in a short cycle time and low cell uptake [99]. To overcome these limitations, many researchers have developed various modified liposomes. Negatively charged mPEG-PLA liposomes have been designed to enhance the affinity to tumor cells due to acidic TME [100]. It is essential to accumulate drugs around the tumor site, which can reduce the side-effects caused by nontarget behaviors. In addition, the synergistic anticancer effects produced by codelivery are of significance for a DDS. The long-circulating CK liposomes coated with targeting peptide ligands that specifically bind to neuropilin-1 receptors on the surface of lung cancer cells have been found to exert an anticancer effect through inducing mitochondrial apoptosis of the A549 tumor cells, damaging the production of the intracellular ROS, as well as reducing the production of the mitochondrial membrane potential, and increasing cytochrome c and Ca^2+^ around the nucleus [101] (Fig. 6).

#### Vesicular nanoparticles

##### Micelles

Micelles are synthesized by amphiphilic surfactant in an aqueous phase, which are promising carriers of ginsenosides for encapsulating drugs with abroad aqueous solubility [119, 120]. The therapeutic effects of ginsenosides in micelles have been investigated in preclinical cancer models, including colorectal and lung cancer cells. Different synthesis methods and anticancer activities of Rh2 and CK micelles have been examined. Amphiphilic PEG micelles modified by celastrol and ginsenoside Rh2 (CG-M) have shown a stronger cell uptake ability, apoptosis induction ability and antiproliferation activity to A549 cells, where the internalization of CG-M to A549 cells was 1.8× stronger than that of free Rh2 [113]. In addition, Solutol® and TPGS have been used to fabricate Rh2-micelles, with an increasing solubility ~ 150-fold greater than that for Rh2, substantially enhancing the antitumor effect [112].

Some researchers have found that CK modified with amphiphilic block copolymer PEG showed sustained release and passive targeting effects on tumor cells. CK polymer micelles (CK-M) with a particle size of 53.07 ± 1.31 nm had good biodegradability and biocompatibility [106]. The growth of A549 and PC-9 cells treated with CK-M were significantly inhibited via blocking the G_1_ phase of the tumor cells. In addition, *bax*, *bcl-2*, MMP-2, *caspase-3* and p-glycoprotein were regulated by CK-M to promote tumor cell apoptosis and inhibit tumor cell invasion, metastasis, and outflow. Because individual polymer micelles have been replaced gradually by binary mixed micelles for their large size and low stability capacity, binary mixed micelles have exhibited higher solubility than individual polymer micelles. Due to their small size and high drug-loading capacity, the intelligent mixed micelles CK PC/PD have higher tumor inhibition through increased permeability and retention effects. Furthermore, the neutrally charged PEG on the micelle surface has reduced nonspecific interactions with blood proteins and increased circulation time [107].

**Fig. 7:**
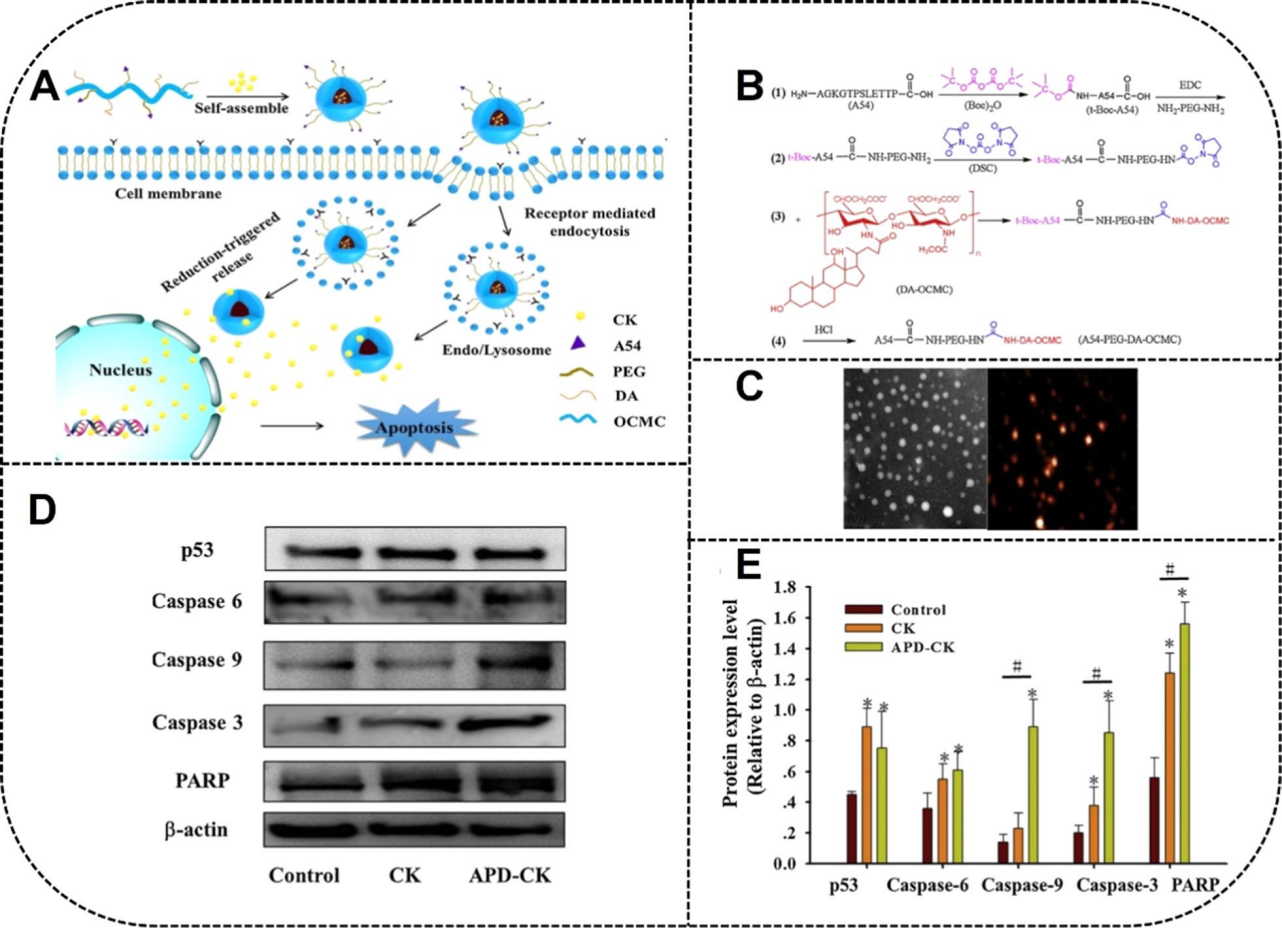
Preparation of A54 peptide-coated micelles and drug delivery of ginsenoside CK to HepG2 and Huh-7 cells. **A** Fabrication and mechanism of APD-CK micelles. **B** Synthetic procedures of A54-PEG-DA-OCMC polymer. **C** TEM image and AFM image of micelles indicating the spherical morphology of APD-CK. **D**, **E** The effect of micelles on the expression of *p53*, *caspase 6*, *caspase 9*, *caspase 3* and PARP apoptosis protein in HepG2 cells. *APD* A54 peptide, *DA* deoxycholic acid, *OCMC* O-arboxymethyl chitosan. Reprinted with the permission from Ref [108]. Copyright © 2020 Elsevier

To further improve the effects of cancer treatment through increasing the accumulation of antitumor drugs at tumor sites and enhancing circulation time, micelles modified with targeting molecules such as peptides can bind to target receptors expressed on the surface of tumor cells to specifically target these tumor cells. Zhang et al*.* have used CS-NPs loaded with CK (CK-NPs) by self-assembly technology, with an average diameter of 171.4 nm [108]. APD-CK coated with peptide A54 may become the potential targeting drug in the treatment of liver cancer with pH-responsive and sustained-release properties under acidic conditions (Fig. 7).

Other CK micelles fabricated by Zhang et al. have induced cell apoptosis and inhibited cell migration by inducing cell cycle arrest in the G_0_/G_1_ phase of A549 cells. In addition, the promotion of apoptosis and the inhibition of P-gp efflux can lead to an obvious antitumor effect of the micelles and an efficient tumor-targeting effect in A549 tumor-bearingmice [109].

##### Transfersomes and ethosomes

Because the traditional liposome therapy remains in the outer lipid layer and cannot penetrate the skin, new types of enhanced liposomes, including transfersomes (TL) and ethosomes (ET), have been developed. TL contains edge activators such as surfactants, which can make the bilayer unstable and increase liposome flexibility. ET is composed of phospholipids, ethanol, and water. The fluidity of the cuticle lipid is increased by the cross-action of alcohol on the lipid bilayer to enhance the drug’s skin penetration.

Choi et al*.* have prepared the vesicles containing ginsenoside Rh1 with an encapsulation rate of 46.77%; the highest encapsulation efficiency of TL was 62.89% and that of ET was 50.49%. TL and ET were treated with Frantz diffusion cells and rat dorsal skin to obtain a skin permeability profile, which showed that Rh1-loaded transfer bodies exerted higher skin permeability as compared with ET and traditional liposomes [19, 121].

#### Microemulsions

MEs are capable of increasing water solubility, enhancing tumor cell absorption, extending retention of NPs in tumor cells, prolonging blood circulation time, and reducing systemic toxicity. Researchers have prepared MEs using different methods [9]. For example, Rg3 MEs and 6′-O-Acetylginsenoside Rb1 MEs have been encapsulated in degradable polylactide (PLA) and poly (dl-lactide-co-glycolide) (PLGA), respectively [102, 104]. Mehrnaz et al*.* have prepared an Rg1 microsphere with degradable poly (propylenefurate) (PPF) by W/O/W secondary emulsification [123]. In addition, Rg3 NPs have been prepared by with 90% (v/v) whey protein isolate aqueous phase and 10% (v/v) medium-chain triglyceride oil phase [22, 124].

As compared with single-component drugs, multicomponent combination therapy can be used to regulate a variety of signaling pathways to achieve efficient tumor therapy. Multicomponent MEs (e.g., ECG-MEs) consisting of etoposide, coix seed oil and ginsenoside Rh2 have been prepared, with a small particle size (73.1 nm) and high encapsulation efficiency (94.3%) [122]. The mechanism of their antitumor effects for A549 tumor xenografts has been found to be related to the small-scale mediated tumor penetration depth and increased serum T helper 1 (Th1) cytokine concentration. Specifically, oral ECG-MEs entered the blood circulation through the intestinal barrier in the form of complete NPs and effectively inhibited P-gp, prolonging blood circulation time and accumulating in the tumor site [114].

#### Protein-based nanocarriers

Proteins increasingly have been researched in functional nanomaterials for preferable absorption, nontoxicity, nonimmunogenicity, and superior stability in vivo. In addition, as compared with other carriers, protein NPs are easier to produce on a large scale. Recently, biological macromolecular proteins based on bovine serum albumin (BSA) [125] and other proteins have been explored to be a potential DDS. Macromolecular proteins can protect the active substance from protein hydrolysis and digestion, enhance the passive drug-targeting property, and prolong circulation life in the blood [126, 127].

An intelligent delivery system with better water solubility based on BSA has been prepared to inhibit cancer. The cytotoxicity experiment in vitro indicated a stronger inhibitory effect on the lung cancer A549, the HepG2 hepatoma, and the HT29 colon cancer cell lines of BSA-CK-NPs [115].

**Fig. 8:**
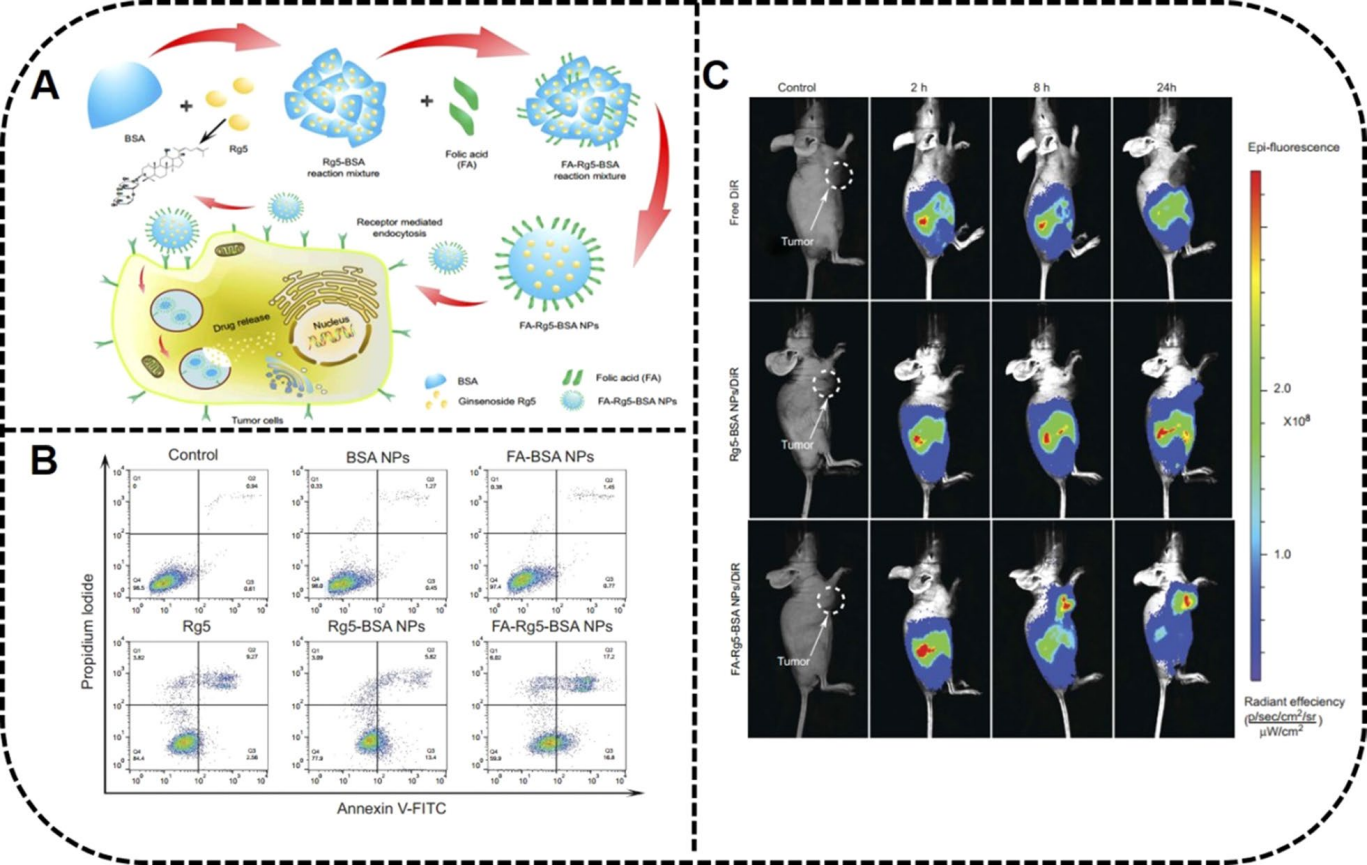
FA-Rg5-BSA NPs to inhibit tumor growth of MCF-7 cells. **A** Preparation of FA-Rg5-BSA NPs and their mechanism to tumors. **B** Cell apoptosis effect of MCF-7 cells treated with Rg5, Rg5-BSA NPs and FA-Rg5-BSA NPs by Annexin V-FITC/PI staining. **C** The in vivo imaging of MCF-7 tumor-bearing mice treated with DiR, Rg5-BSA NPs/DiR, and FA-Rg5-BSA NPs/DiR. Reprinted with the permission from Ref [118]. Copyright © 2019 Dove Medical Press

Folic acid (FA) is a targeting moiety in anticancer drug delivery, partly due to the high expression of folate receptors in tumor cells [128]. Dong et al*.* have prepared BSA-NPs modified with ginsenoside Rg5 and folate for a targeted tumor treatment. The EPR effect and receptor-mediated targeting were the main reasons for MCF-7 cell apoptosis. Furthermore, Rg5 released from FA-modified targeted NPs were efficiently accumulated within 8 h at the tumor site in a MCF-7 xenograft mouse model [118] (Fig. 8).

Albumin, the main source of amino acids and energy in solid tumors, has been applied to recognize the protein binding receptor glycoprotein 60 (gp60), which mediated endocytosis on the surface of tumor cells [129]. In addition, various functional groups exist on the surface of albumin. Further specific targeting can be achieved by modifying the corresponding ligands to improve the pharmacokinetic and tissue distribution properties. The modification of albumin not only has achieved specific targeting, but also has endowed albumin with other characteristics, such as increasing the stability of albumin NPs and prolonging the half-life in vivo. However, the albumin NPs remained unstable enough due to the various enzymes and proteins of the complex environment containing in vivo. The recombination of disulfide bonds between albumin molecules can not only reassemble these molecules into comparatively stable NPs but also can endow the drug with a property of reduction responsive release by glutathione in the TME [130]. Shortcomings remain for protein drug delivery, such as degradation by enzymes and short half-life, which can result in the reduced uptake of NPs by tumor cells. However, the surface-modified proteins were capable of alleviating the defects, for instance, the hydrophobicity of albumin can be increased by laurylamines, which can reduce the nonspecific phagocytosis of RES and increase the stability of NPs [131].

#### Metallic and inorganic nanoparticles

Metal and inorganic NPs have been reported widely for the advantages of their large specific surface area, easy surface modification, strong stability, and high drug-loading rates [132–136]. For example, gold nanoparticles (GNPs) are widely used in biomedical platforms due to their easy preparation, surface modification, and optical properties. Furthermore, the carbon dots in carbon nanomaterials have unique fluorescence properties, and carbon nanotubes (CNTs) and graphene oxide (GO) have a high drug loading rate. In addition, mesoporous silica (MS), as a kind of DDS, has a large specific surface area and nontoxic properties.

##### Gold nanoparticles

GNPs have emerged as a prominent delivery vehicle due to their large specific surface area, easy modification, multimodal imaging agent, and good biocompatibility in biological applications, making them multifunctional nanocarriers with biodegradability and photothermal therapy (PTT) after laser irradiation. Because of gold’s high binding affinity of gold with sulfhydryl and amino groups, gold nanoparticles, with the property of being functionalized easily by antibodies, proteins, nucleic acids, and carbohydrates, successfully have achieved selective targeting to tumor tissues [137].

**Fig. 9:**
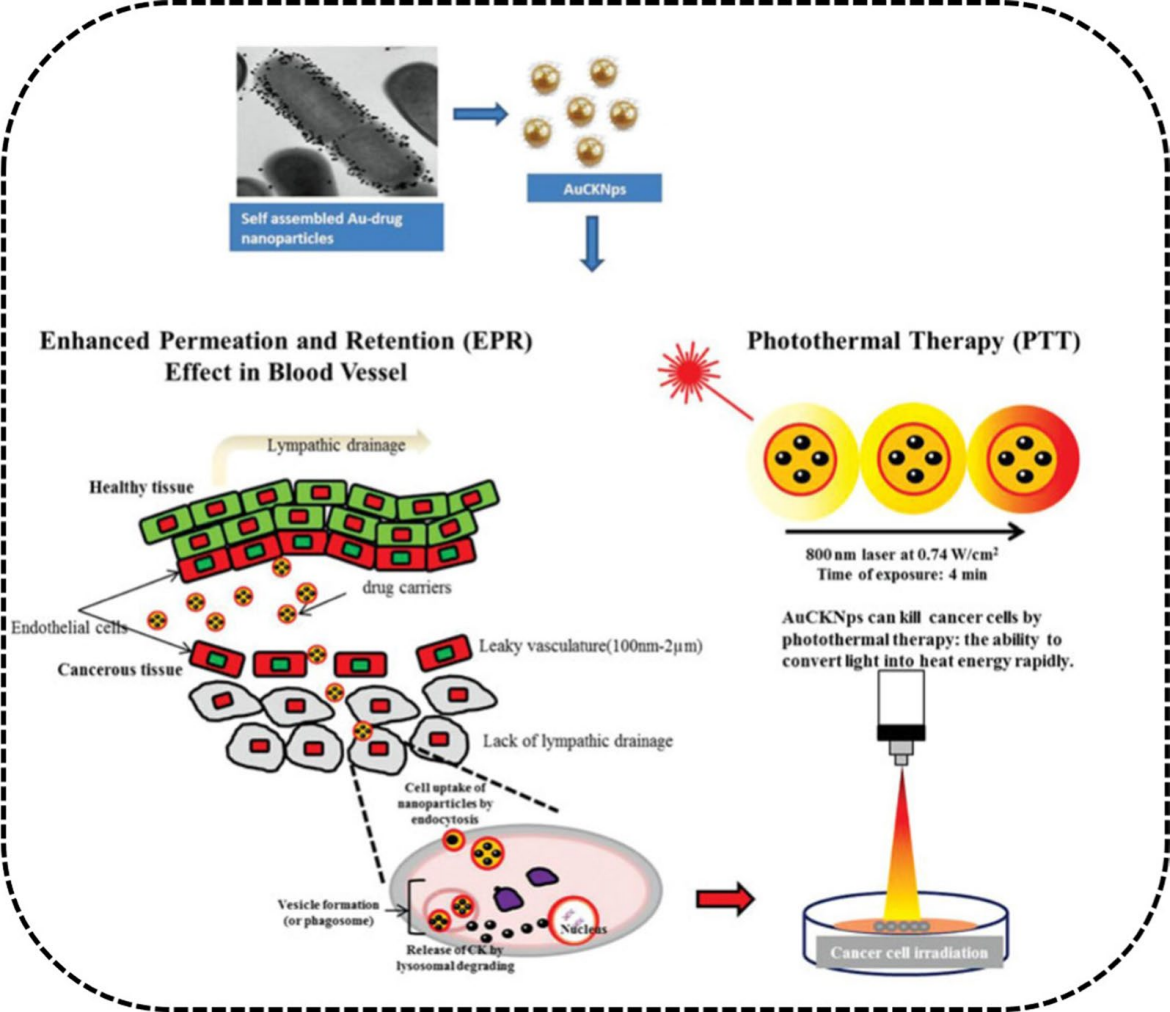
Mechanism of A549 cells and HT29 cells internalization of DCY51^T^-AuCKNps by EPR effect. PTT of AuCKNps by convert light into heat rapidly. Reprinted with the permission from Ref [110]. Copyright © 2019 Taylor & Francis

Ginsenoside CK-GNPs with pharmacological activity have been verified as potential nanocarriers of ginsenoside CK [32]. Furthermore, probiotic lactobacillus DCY51^T^ has been utilized to fabricate ginsenoside CK-GNPs (DCY51^T^-AuCKNps) using the one-pot method [110]. In addition to its good optical properties, DCY51^T^-AuCKNps can provide PTT with a laser, which exhibited synergistic chemotherapy effects on the human lung adenocarcinoma cell line A549 and human colorectal adenocarcinoma cell line HT29. Photodynamic therapy (PDT) also has been investigated widely in cancer therapy with GNPs [138] (Fig. 9).

##### Carbon nanomaterials

As compared with traditional drug carriers, carbon nano drug DDSs have displayed more competitive merits such being inexpensive and easy to obtain, as well as having a large specific surface area, easy functional modification, and better excretion and degradation properties [139]. Carbon-based nanomaterials, including CDs, CNTs, and grapheme, have been used widely in the biomedical field due to their unique size and properties [140–142].

CDs are zero-dimensional nanomaterials with light stability, excellent photoluminescence quantum yield, and photo-bleaching resistance. When compared with heavy metal nanomaterials, CDs exhibit better biocompatibility and lower toxicity. The small-sized Re-CDs exploited by Yao et al. have been shown to be beneficial to cellular uptake, which inhibited tumor cell proliferation through the ROS-mediated pathway, thus inducing cell apoptosis [117]. CNTs, as an allotrope of carbon, are one-dimensional quantum materials that can be conjugated with drugs on their surface, further enhancing the drug delivery and targeting abilities [143, 144]. Rb-CNT and Rg-CNT DDSs using CNTs as drug carriers were designed for antiproliferation of breast cancer (MCF-7) and pancreatic cancer (PANC-1) cells [103].

GO, is a layer of tightly arranged carbon atoms combined with hexagonal honeycomb lattice with a highly specific surface area, easy surface modification, and anticancer activities [145–147]. GO-Rh2 has shown higher antitumor activity and the lowest toxicity to the coagulation system and heart tissue for the functionalization of the positively charged amino acids lysine (Lys) and arginine (Arg) [116, 148].

The strong photothermal absorption capacity of CNTs and GO can be utilized for PTT. The combination of PTT and chemotherapy can not only obtain higher therapeutic effects than can PPT or chemotherapy alone but also can reduce chemotherapy’s side-effects. Interestingly, GO is superior to CNTs in PTT due to its smaller size, better dispersion and optical advantages [149].

##### Mesoporous silica

MS is an important drug delivery nanocarrier with a large specific surface area, strong stability, degradability, high drug-loading rate, and nontoxic properties [150, 151]. The silanol hydroxyl group on the surface and adjustable pore size are beneficial for the combination of various drug molecules. Singh et al*.* have loaded ginsenoside CK and Rh2 in 200 nm of porous silica, which exerted an excellent biocompatibility with normal hacaT skin cells and anticancer effects on HepG2, A549, and HT-29 colon cancer cells [111]. Because MS has merits such as chemical stability with the Si–C bond, stimuli-responsive molecular gates, and a degradable carrier [152], a variety of responsive organic or inorganic stimuli-responsive molecular gates can be added to the surface of silanes to create a chemical modification for controlling drug release.

### Ginsenosides as carriers

#### Liposomes

Ginsenosides have shown a potential role in stabilizing the phospholipid bilayer, with a structure and properties similar to those of cholesterol [20, 26]. As compared with cholesterol, ginsenosides, as both adjuvant drug and excipient, have many merits, including not only enhancing the stability of liposomes and prolonging circulation time but also displaying active targeting and cooperation with chemotherapy drugs. The idea of ginsenosides replacing cholesterol was put forward by Ajay et al*.* and confirmed by Wang et al*.* [19], which also has been verified with pin labeling and paramagnetic resonance [26].

Ginsenosides can not only regulate the orderly arrangement of the phospholipid bilayers to increase stability but also can regulate the hydrophobicity of the liposome membrane. Rg5-paclitaxel (PTX) liposomes proposed by Li et al*.* have been confirmed with HGC-27, A549 and MCF-7 subcutaneous tumor models, which achieved curative effects by through targeting the GLUT receptor on the tumor surface [27]. A multifunctional liposome of ginsenosides Rh2, Rg3, and Rg5 combined with PTX have confirmed that the DDS with a combination of drugs of different structures can provide combination therapy.

As a result, ginsenoside liposomes are prone to be accumulated in tumors because of their ability to recognize the GLUT carrier on the tumor cell membrane with stronger toxicities and targeting abilities to BGC-823 cells as compared to the cholesterol liposome. Research has verified that Rh2, Rg3, and Rg5-liposomes were mainly taken up through the GLUT1 and SGLT1, as well as the GLUT5 or the GLUT2 pathways [26]. In addition, the PTX-Rh2-liposome achieved excellent tumor-targeting and antitumor activity in a mouse breast cancer model [25]. From these investigations, ginsenosides not only exerted their inherent antitumor activity but also showed a significant synergistic effect with PTX.

**Fig. 10:**
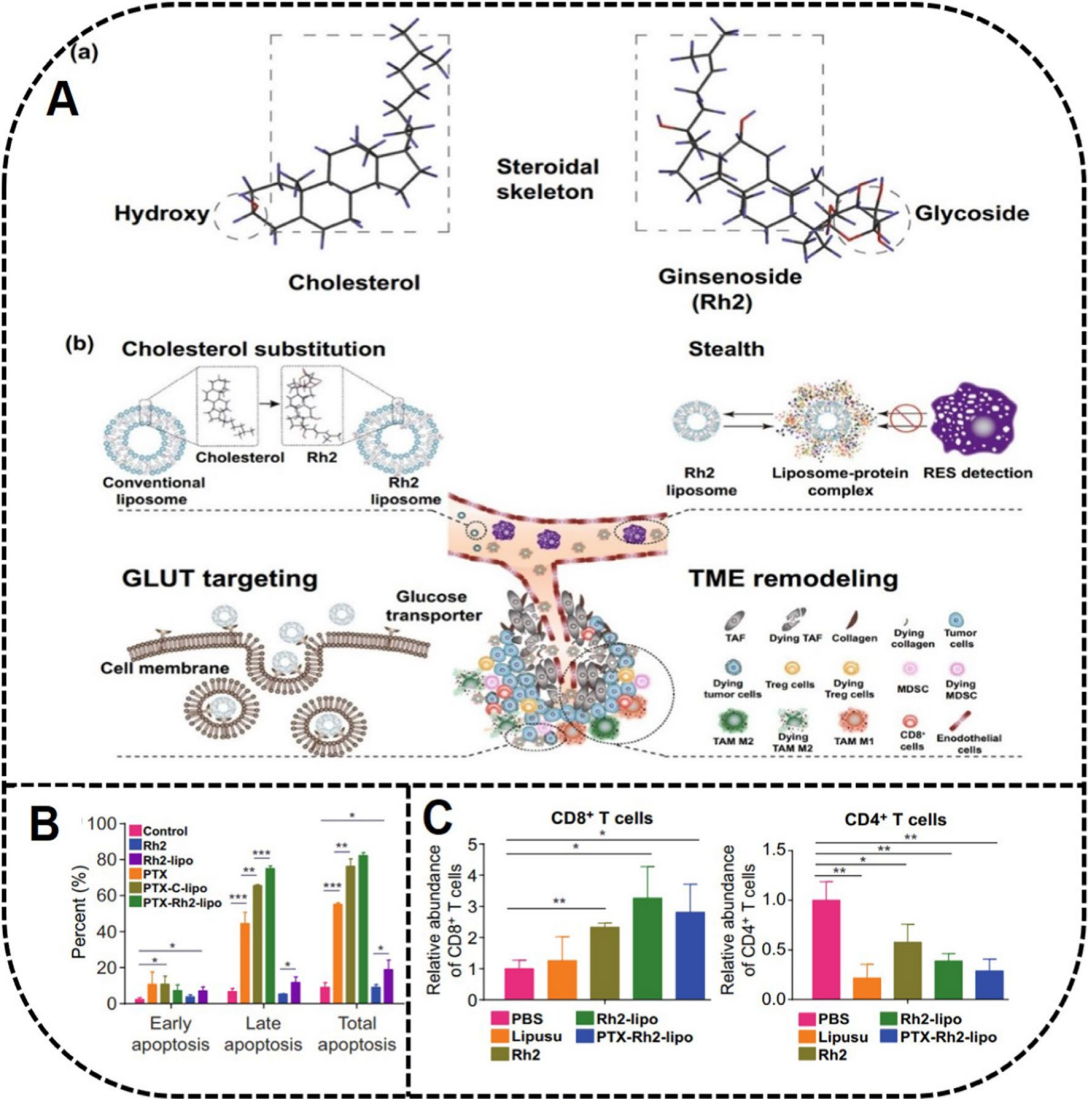
In vivo antitumor activity by PTX-Rh2-liposome. **A** Fabrication and application of PTX-Rh2-liposome. **a** The similar structure of ginsenoside Rh2 and cholesterol. **b** The properties including membrane stability, stealth, GLUT targeting to tumors, as well as TME remodeling of ginsenoside Rh2. **B** PTX-Rh2-liposome induced apoptosis of tumor cells. **C** The increased CD8^+^ T cells and decreased CD4^+^ T cells induced by PTX-Rh2-liposome. Reprinted with the permission from Ref [25]. Copyright © 2020 Springer

Cholesterol and PEG-C liposomes can decrease the elimination rate of liposomes mainly by reducing the affinity and adsorption capacity of Ig. PTX-Rh2 liposomes prepared by Hong et al*.* have shown a long-circulating role by effectively reducing the adsorption of opsonins on the surface of the liposomes, and increased the adsorption of apolipoprotein E, which can retard the absorption of macrophages to liposomes [25] (Fig. 10), because the opsonins, including Ig and complement proteins are the main targets of RES. The stealth effect of Rh2 liposomes reduced RES clearance by decreasing the adsorption of opsonins.

**Fig. 11:**
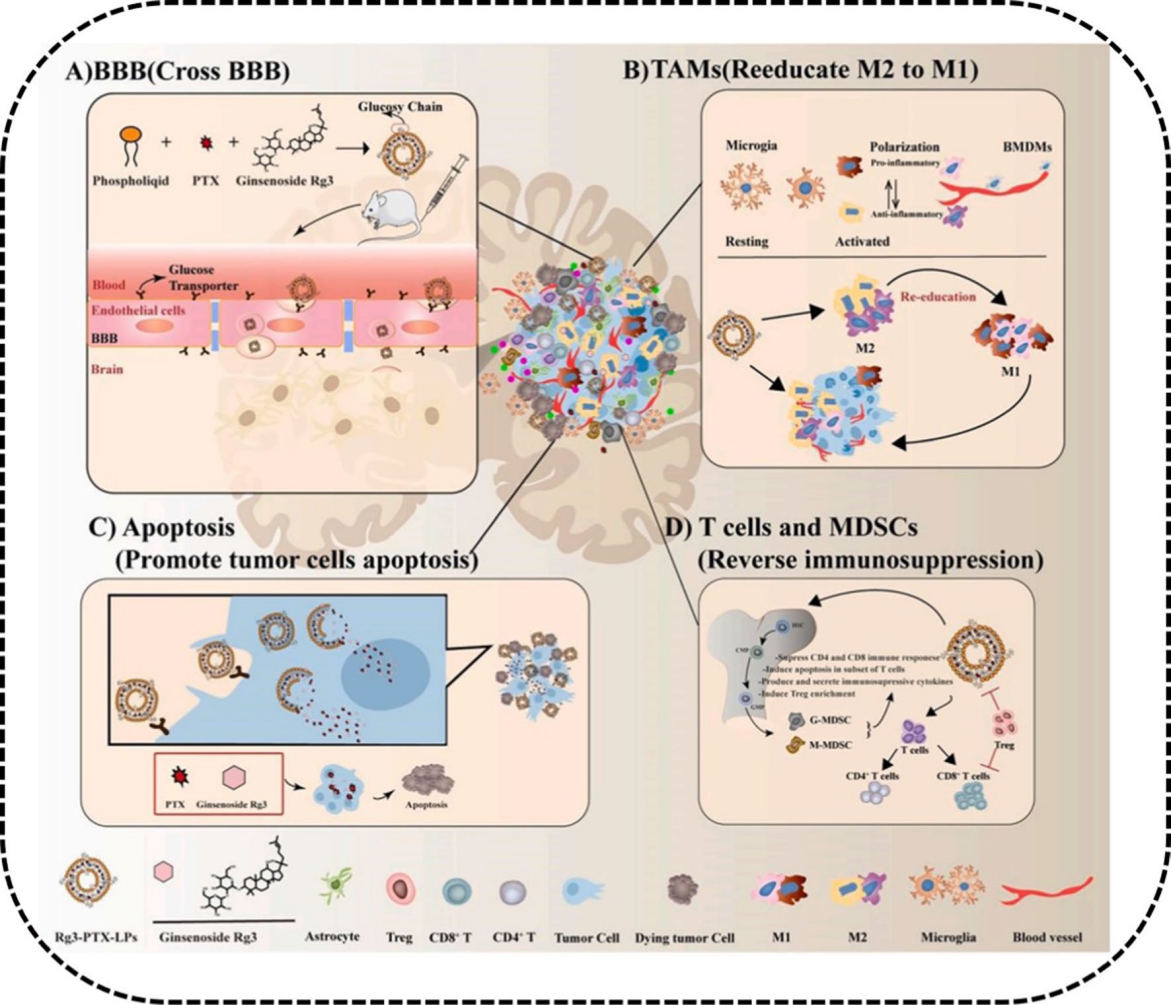
Multifunctional activities of Rg3-PTX-liposomes in vivo. **A** Rg3 substituting cholesterol are utilized to fabricate Rg3-PTX-liposomes. The liposomes recognize GLUT of BBB through the glycosyl moiety of Rg3 and are easier to penetrate to brain across the BBB. **B** Rg3-PTX-liposomes targeting to TAM induce the transformation of M2 into M1, thus stimulating tumor immunotherapy. **C** Rg3-PTX-liposomes induce apoptosis of tumor cells. **D** Rg3-PTX-liposomes promote the differentiation of T cells into CD4^+^ and CD8^+^T cells. Reprinted with the permission from Ref [28]. Copyright © 2021 Elsevier

Furthermore, the immunotherapeutic activity of ginsenoside liposomes has been reflected in the improvement of the TME structure and the changing of the immune-deficiency TME. This improvement occurred mainly through reducing the heterogeneous cells in the TME and increasing the infiltration of the CD8^+^ T cells [25], as well as inhibiting tumor proliferation by activating the C6 glioma immune microenvironment via inducing the transformation of the M2 TAMs into M1 in TME [28] (Fig. 11).

#### Biomimetic nanoparticles

A majority of delivery systems can provide improvements in various characteristics by improving the drug loading rate, water solubility, and drug targeting [153]. It would be useful for modified NPs to increase the blood circulation time and target effect, so as to achieve the effects to reduce toxicity and increase efficiency [83]. However, some limitations remain in the clinical application of common nanomaterials.

For example, as substances of external origin, NPs are likely to be recognized and eliminated by the immune system, which creates the common problem of high immunogenicity in vivo [154]. Therefore, biological DDSs (BDDs) have been used widely in disease diagnosis and treatment due to their high biocompatibility, long-circulating time, targeting effects, and low immunogenicity. Biomimetic materials, including natural product carriers; nutrient transporter ligands abundantly expressed on the surface of tumor cells, microneedles; and cell membranes from red blood cells, leucocytes, and platelets, have been used extensively to fabricate BDDs [155–158].

Ginsenosides are amphiphilic molecules with the glycosyl hydrophilic group at the C-3 position and the hydrophobic group at the C-17 position, which exhibit the potential function of stabilizing the phospholipid bilayer as carriers. Some ginsenosides have a structure with a cholesterol-like steroidal mother nucleus. Several studies have proved that cholesterol, as one of the ideal components of liposomes, improved liposome efficacy and the stability of the liposome membrane [159, 160]. The liposomes containing cholesterol available by prescription in the marketplace have shown certain antitumor effects; however, cholesterol has shortcomings, such as hyperlipidemia, pulmonary hypertension, and other diseases caused by the excessive absorption of cholesterol by the human body [75, 76]. In addition, the high content of cholesterol in TME is closely related to tumor growth.

Different from cholesterol, ginsenoside-encapsulated liposomes have been extensively investigated for their anticancer properties [26]. Liposomes encapsulated with PTX have been shown to produce synergistic anticancer effects [97]. New liposome-delivery technology has exhibited the potential application prospects for using ginsenoside instead of cholesterol-encapsulated liposomes in pharmacological applications (Table 5) [26, 161–165].

**Table 5:** Cholesterol-liposomes in the marketplace and ginsenoside-liposomes in research for tumor therapy

Formulation	Product	Prescription (mole ratio)	Application	References
Doxorubicin HCl liposomes injection	Doxil®	HSPC/CHOL/DSPE-mPEG2000 (3:1:1)	Ovarian cancer; metastatic breast cancer; multiple myeloma	[161, 162]
Vincristine sulfate liposomes injection	Marqibo®	SM/CHOL (3:2)^a^	Acute lymphoblastic leukemia; refractory cancer	[163, 164]
Irinotecan hydrochloride liposome injection	Onivyde®	DSPC/CHOL/mPEG2000-DSPE (430:285:3)^b^	Pancreatic cancer	[165]
PTX liposomes	NA	EYPC/Rh2 (10:3)	4T1 breast carcinoma	[25]
PTX liposomes	NA	EPC/Rg3 (10:3)	C6 murine glioma cells	[28]
PTX liposomes	NA	EYPC/Rh2 (10:3)	Gastric cancer	[26]
PTX liposomes	NA	EYPC/Rg3 (5:2)	Gastric cancer	[26]
PTX liposomes	NA	EYPC/Rg5/soybean oil (10:4:5)	Gastric cancer	[26]
PTXginposome	NA	Lecithin/Rg5 (5:2)	HGC-27; A549; MCF-7	[27]

*PTX* paclitaxel, *HSPC* hydrogenated soybean phosphotidylcholine, *CHOL* cholesterol, *SM* sphingomyelin, *DSPC* distearoyl phosphatidylcholine, *DSPE* distearoyl phosphoethanol- amine, *EYPC* egg yolk lecithin, *NA* not applicable, *a, b* prescribing information from medicine instruction

Wang et al*.* have developed PTX liposomes with Rh2, Rg3 and Rg5 instead of cholesterol. The ginsenoside liposomes not only exerted their inherent antitumor activity by targeting the GLUT on the tumor surface, but also showed significant synergistic effects with PTX by effectively reducing the adsorption of opsonins on the surface of the liposomes, thus improving the structure of the TME, as well as reversing the state of tumor immunodeficiency [26].

**Fig. 12:**
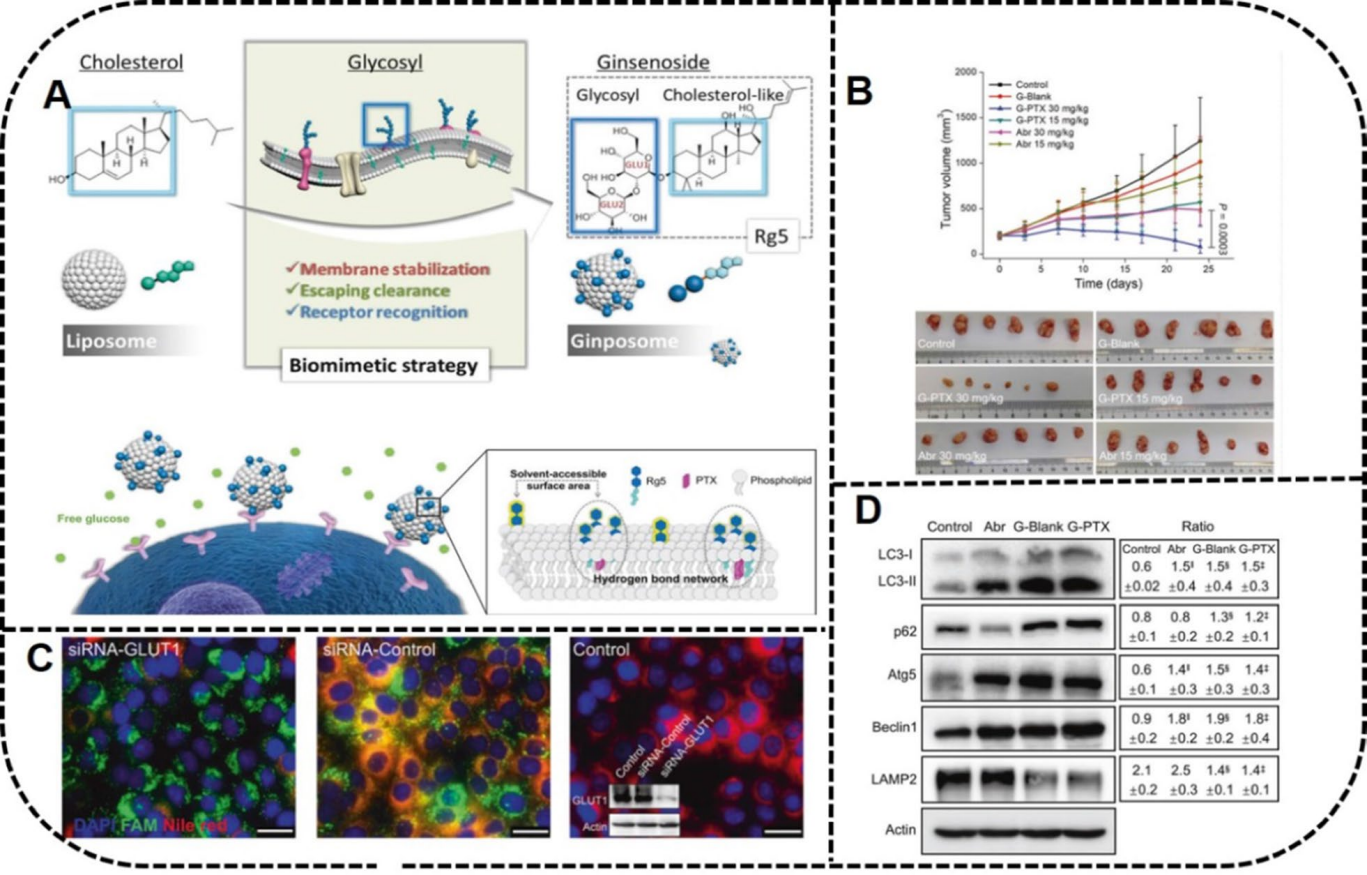
In vivo antitumor activity by G-PTX. **A** Fabrication of ginsenoside-anchored liposome and surface glycosyl of ginposome for active-targeting ability to GLUT1 receptor on the surface of tumors. **B** The suppressed tumor growth of patient-derived xenograft tumor models treated with G-PTX. **C** The verification of G-PTX active targeting, G-PTX uptake was reduced via inhibiting GLUT1 by siRNA transfection. **D** The expression of autophagy-related proteins of HGC-27 cells indicating the reversal of drug resistance. Reprinted with the permission from Ref [27]. Copyright © 2021 Springer Nature

The concept of using a ginsenoside anchored liposome (ginposome) was first proposed by Li et al. The encapsulation efficiency of the PTX-loaded ginposome (G-PTX) was 97.2%, with a spherical particle size of 110 nm. In addition, the biomimetic property of Rg5 was demonstrated by the glycosyl which was exposed on the surface of the liposomes. Rg5 was screened as the most ideal cholesterol substitute through a molecular dynamics simulation. It also was verified that the long circulation time of G-PTX was attributed to the third skeleton of the disaccharide group. Furthermore, the broad-spectrum targeting ability of G-PTX was confirmed by the HGC-27, A549, and MCF-7 subcutaneous tumor models, which achieved a curative effect through targeting the GLUT receptor on the tumor surface, and was confirmed further through the clathrin and caveolae-dependent pathways for endocytosis [27] (Fig. 12).

Using ginsenosides simultaneously both as an adjuvant drug and as an excipient have been investigated in depth. Ginsenoside-endowed liposomes used as an excipient have a long cycle targeting function, which can greatly improve the drug delivery efficiency. The formulations for the preparation of liposomes were simple without adding PEG and targeting ligand, which simplified the process of technological production. In addition, ginsenosides can reduce the immunogenicity of the liposomes, and reverse the state of TME immune deficiency by their biomimetic properties. Thus, the synergistic anticancer effects with chemotherapeutic drugs were achieved [25–27]. The new liposome-delivery technology-ginposome can inspire the design of additional DDSs. The technology also is in clinical transformation, which is of great significance for tumor treatment.

According to current research, the biomimetic characteristics of ginsenosides are reflected in three aspects [25–28]. First, as the main targets of RES, the adsorption of opsonins including Ig and complement proteins which are the main targets of RES, can be reduced effectively on the surface of the liposomes. Therefore, liposomes modified with ginsenosides exhibited a stealth effect and increased the adsorption of apolipoprotein E, which can retard the absorption of the macrophages Second, the immunotherapeutic activities of the ginsenoside liposomes were reflected in improving the TME structure to enhance the drug permeability by reducing the tumor vascular density and destroying the expression of tumor-associated fibrocyte and collagen cells, which displayed a protective effect on the tumor cells. Finally, ginsenoside liposomes changed the immune-deficiency TME, mainly through reducing the heterogeneous cells in the TME and enhancing the immune function by increasing the infiltration of the CD8^+^ T cells.

Furthermore, ginsenosides can be utilized to target TAM2 while the antitumor drugs play a role in reconstructing the TME by transforming TAM2 into TAM1 to promote the role of the T cells [25]. Ultimately, the NPs enhanced the tumor-killing and prognosis effects through the combination of immunotherapy and drug therapy. The biomimetic properties are worthy of further investigation to expand the application value of ginsenoside in liposome research, while also exploring the application of effective components of TCM. Although PTX and gemcitabine already have made some progress in biomimetic drugs [155], the research on ginsenoside biomimetic drugs is still in the early stages. Therefore, the exploration of ginsenoside codelivery of biomimetic nanomaterials provides a new idea for the clinical application of a ginsenoside DDS, with a good research and application prospects.

## Encapsulated ginsenosides improve biological functions

### Pharmacokinetic properties

Ginsenosides, with their variety of important bioactivities, can resist diabetes, depression, and cancer and also exhibit better protective effects on cerebral ischemia, endothelial cell injury, and CVD [42–44]. Research on the pharmacokinetics of drugs, including absorption, distribution, metabolism, and excretion, plays an important role in comprehending the pharmacological and toxicological effects of drugs on the body [11, 17, 166]. Furthermore, understanding the pharmacokinetic parameters is beneficial for avoiding adverse reactions and determining the appropriate dosage of drugs, as well as for planning a dosage regimen and improving clinical efficacy.

The multiple pharmacokinetic properties of different DDSs have been discussed because those of ginsenosides can reflect the efficacy and toxicity of the drugs [35]. Nao-Qing ME could be injected into the organs or tissues through blood circulation by intranasal or intragastric administration. Concentrations of the active components Rb1 and Rg1 from Nao-Qing ME were linear in pharmacokinetics after intranasal or intragastric administration, then reaching the brain, heart, liver, lungs, and kidney, respectively.

The nasal mucosa is rich in blood vessels, which is conducive to drug circulation into the body. Therefore, the highest concentration of Rg1 was achieved by intranasal administration after 5 min, while it was achieved by intragastric administration after 1 h. Intranasal administration greatly shortened the T_max_ of Rg1, while increasing the C_max_ and prolonging the half-life of Rg1 (7.9127 h vs. 56.1723 h). In addition, it greatly increased the area under the concentration curve (AUC) in the rat brain. These results indicate that intranasal administration can promote the absorption of Rb1 and Rg1 [167], because it avoids the BBB and moves directly through the cerebrospinal fluid to the brain.

Zhao et al*.* have developed CK NPs (CK-SSD) modified with self-nanomicellizing solid dispersion (SSD). As compared with free CK, the AUC of CK-SSD was increased by 2.02×, which indicated the improved bioavailability of drugs with solid dispersion (SD) [168]. Yu et al*.* have measured the pharmacokinetic parameters of the ginsenoside Rg3 liposome (L-Rg3) by intravenous injection, with a half-life of ~ 30 min. The C_max_ and AUC values of the liposomes were increased by 1.19× and 1.52× ,t respectively. Furthermore, their effects for enhancing the permeation and retention of the lungs and liver promoted the absorption of drugs, showing that liposomes can improve the anticancer activity of clinical drugs [97]. The AUC values of Rg1 and Rb1 in a phospholipid complex increased by ~ 15× and 6×, respectively [169].

It is crucial for nanocarriers to improve drug bioavailability. In contrast to oral and intragastric administration, ginsenosides were widely distributed in the body after intravenous and intranasal administration, then passed through the BBB. The pharmacokinetic parameters of ginsenosides and their delivery system in rats are shown in Table 6. To date, many ginsenoside delivery systems have been investigated, but only a few researchers have examined their pharmacokinetics of delivery system. The in-depth study of pharmacokinetics can reveal their biological function and mechanism in vivo. Additional research is necessary, because good pharmacokinetic properties are the prerequisite for the application of nanodrugs in cancer treatment.

**Table 6:** Pharmacokinetic parameters of ginsenosides and its formulations in rats

Dosage	t_1/2_	C_max_	T_max_	V_d_/F	CL/F (CL)	AUC_0-∞_	MRT	Bioavailability	RefS
Nao-Qing emulsion 0.6 mg/kg (Rg1, pr.nar.)	56.1723 h	16.65 μg/mL	0.08 h	0.082	0.0443	592.92 μg·h/mL	17.12 h	NA	[167]
Nao-Qing emulsion 0.6 mg/kg (Rg1, ig)	7.9127 h	11.29 μg/mL	1.00 h	0.0673	0.0668	101.70 μg·h/mL	12.86 h	NA	[167]
CK-SSD 35 mg/kg (po)	4.8 ± 2.5 h	518.1 ± 185 μg/mL	0.4 ± 0.1 h	NA	NA	2434.2 ± 2008.3 μg·h/L	6.8 ± 1.6 h	NA	[168]
CK 35 mg/kg (po)	5.4 ± 0.8 h	253.6 ± 143.3 μg/mL	3.0 ± 0 h	NA	NA	1203.1 ± 636.6 μg·h/L	6.7 ± 0.8 h	NA	[168]
PNS-complex (Rg1, po)	NA	NA	NA	NA	NA	27.38 μg·h/mL	NG	50.56%	[169]
PNS-complex (Rb1, po)	NA	NA	NA	NA	NA	600.08 μg·h/mL	NG	59.49%	[169]
Liposomal Rg3 0.5 mg/kg (iv)	0.491 h	3343.05 μg/mL	NA	NA	34.266 L/h/kg (CL)	583.676 μg·h/L	0.184 h	NA	[97]
Rg3 0.5 mg/kg (iv)	0.540 h	2185 μg/mL	NA	NA	52.081 L/h/kg (CL)	384.02 μg·h/L	0.196 h	NA	[97]

*CK-SSD* compound K self-nanomicellizing solid dispersion system, *PNS-Complex* Panax notoginseng saponins, *NA* not applicable

### Pharmacological activities

In recent years, the mechanisms of ginsenosides have attracted much attention. These mechanisms have been verified by extensive research, mainly through inhibiting tumor cell proliferation, invasion and metastasis; inducing tumor apoptosis; reversing tumor cell multidrug resistance; and changing the tumor immune-deficiency microenvironment. With a ginsenoside DDS applied to various tumor cells with different antitumor mechanisms, the G_0_/G_1_ phase of cancer cells can be inhibited by a CK delivery system with an intervening and regulating cell cycle to inhibit the proliferation of tumor cells [109]. In addition, CK micelles and liposomes can promote tumor cell apoptosis by regulating *bax, bcl-2* and *caspase-3* of lung cancer cells A549 and PC-9, which can induce mitochondrial apoptosis and damage ROS production [101, 106]. The CK micelles also can promote tumor cell apoptosis by regulating *caspase-3, caspase-9* and the ADP ribose polymerase proteins of the liver cancer cells HepG2 and Huh-7.

Rg3 micelles and liposomes can promote apoptosis by increasing *caspase-3* [88]. Both can inhibit tumor proliferation by reducing the expression of PCNA and MVD [97]. In addition, invasion and metastasis, the characteristics of malignant tumors can result in the treatment failure for invasion and metastasis. MMPs can destroy the histological barrier of tumor cell invasion, creating conditions for tumor invasion and metastasis. A CK delivery system can reduce MMP production and inhibit tumor cell migration and outflow [106]. Furthermore, Rh2 micelles and liposomes can enhance the therapeutic effects of drug-resistant breast cancer by inhibiting CYP450-mediated metabolism and P-gp efflux [9].

### Multifunction as drugs

**Fig. 13:**
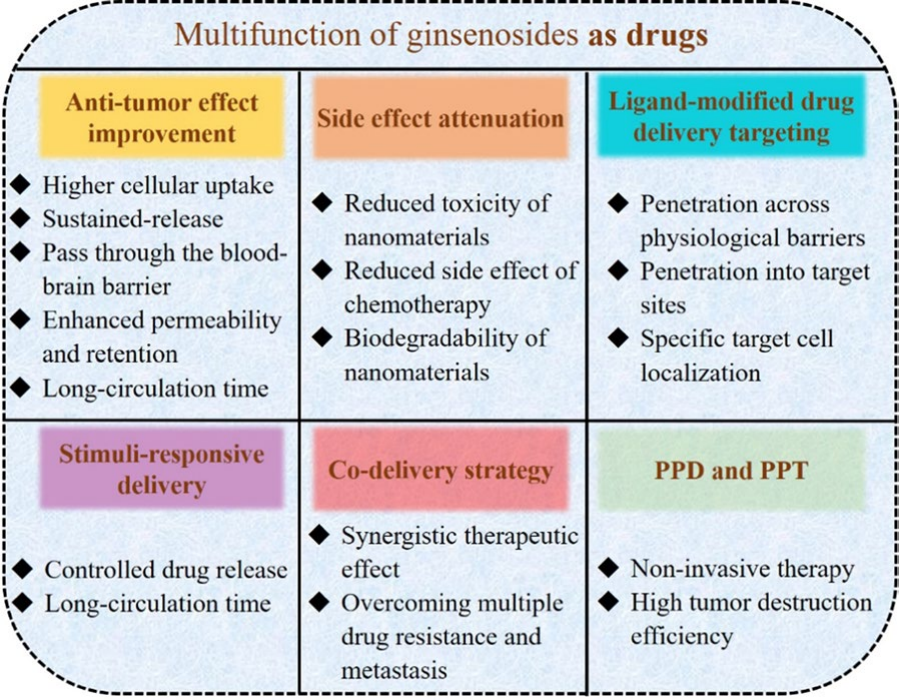
Multifunction of ginsenosides as drugs

Ginsenosides, as bifunctional drugs, have shown promising multiple pharmacological functions, which are summarized in Fig. 13 including antitumor effect improvement, side-effect attenuation, ligand-modified drug delivery targeting, stimuli-responsive delivery, and photodynamic or photothermal therapy enhancement.

#### Antitumor effect improvement

The types and charges of ligands on its surface, as well as the particle sizes of delivery systems, can affect the interaction of nanomedicines with cells and other components in blood including inhibiting the phagocytosis of macrophages and diminishing RES uptake. These can affect ginsenoside penetration and uptake in vivo.

In one study, different hydrophilic carriers such as PEG, CS, amphiphilic block copolymers, polypeptides, and proteins, were loaded on the surface of NPs to shield the phagocytosis to achieve an antitumor effect with sustained-release and long circulation time. The authors have confirmed that ginsenoside delivery systems with carriers can pass through the BBB and enhance the penetration and retention of drugs in tumors. PEG is the most widely used nonionic hydrophilic polymer with solubilization and stealth properties [170]. In addition, ligands including PEG, CS, amphiphilic compounds, peptides and proteins can reduce the tendency of particle aggregation through spatial stability, thus exhibiting higher stability in storage and application [88, 90, 96, 101, 118].

Due to the binding of ligand molecules, the pharmacokinetics of the drugs have changed, resulting in prolonged blood circulation time. The possibility of the drugs reaching the site of action can be enhanced by their avoiding being identified and removed from the body. PEGylated drugs and nanocarriers have been characterized by reduced renal filtration, enzymatic degradation and RES uptake. Therefore, the drug modified by ligands showed a prolonged half-life in vivo, and the drug combined with CS and peptide also can pass through the BBB, thus improving bioavailability. APD-CK micelles modified with targeting peptide A54, which specifically binds to liver cancer, might become the potential targeting drugs in the treatment of liver cancer with pH-responsive and sustained-release properties under acidic conditions. In addition, it has been confirmed that ginsenoside Rg3 modified with amino acids, peptides, and CS can pass through the BBB and prolong the blood circulation time of drugs in vivo.

Among them, the amino acid (mPEG-b-P (Glu-co-Phe)) NPs can target cancer cells owing to pH sensitivity and have a longer circulation time in the blood [88]. In addition, ANG-Rg3-NP modified with vascular endothelial cell-2 (angioep-2) polypeptide has shown good sustained-release behavior and inhibited the proliferation of C6 glioma cells in a concentration-dependent manner, while angioprep-2-functionalized NPs crossed the BBB more easily and accelerated their cell uptake [89]. Furthermore, the fluidity of the cuticle lipid was increased by the cross-action of alcohol on the lipid bilayer to enhance the skin penetration of the drug. Therefore, TL and ET containing ginsenoside Rh1 exerted higher skin permeability compared with traditional liposomes [19, 121].

Delivery systems with different charges can achieve antitumor effects with increased permeability and retention. Neutral or negatively charged particles have shown a longer blood circulation time, while positively charged particles have shown higher cellular uptake.

On the one hand, the neutrally charged PEG on the micelle surface reduced nonspecific interactions with blood proteins and increased circulation time [107]. Furthermore, the negatively charged anions on the surface of liposomes Rh2-PLP enhanced the affinity to tumor cells in the acidic TME to avoid RES [100]. In addition, GO-Rh2-NPs with a weak positive charge can interact with the tumor cell membrane due to the anionic lipid on its surface, which showed higher antitumor activity and the lowest toxicity to the coagulation system and heart tissue for the functionalization of positively charged amino acids Lys and Arg [116, 148].

On the other hand, the PTX-Rh2-liposome played a long-circulating role by effectively reducing the adsorption of opsonins on the surface of liposomes, and increasing the adsorption of apolipoprotein E, which can retard the absorption of macrophages to liposomes [25]. The stealth effect of the Rh2 liposome reduced RES clearance by decreasing the adsorption of opsonins which, including Ig and complement proteins, are the main targets of RES.

Particles with small sizes can increase tissue permeability, but the stability of NPs in the blood circulation could be reduced to a certain extent [171]. The small-sized Re-CDs were beneficial to cellular uptake, which inhibited tumor cell proliferation through the ROS-mediated pathway, thus inducing cell apoptosis [117]. To further improve the effects of cancer treatment through increasing the accumulation of antitumor drugs at tumor sites and enhancing circulation time, multicomponent ME consisting of etoposide, coix seed oil, and ginsenoside Rh2, with a small particle size (73.1 nm), entered the blood circulation through the intestinal barrier in the form of complete NPs and inhibited P-gp, then prolonged circulation time and accumulated in the tumor site [122].

Targeting NPs and microenvironment-sensitive NPs can reduce the toxicity and side-effects of drugs. Targeted DDSs can cross physiological barriers, penetrate into target sites, and specifically target cell localization with reduced toxicity and longer circulation time in the blood [172, 173]. In addition, ginsenosides can provide improvements in tumor immunotherapy by reconstructing TME. The stealth effect of the Rh2 liposome reduced RES clearance by decreasing the adsorption of opsonins, the main targets of RES. Furthermore, the immunotherapeutic activity of the Rh2 liposome also has been reflected in improving the TME structure and changing the immune-deficiency TME, mainly through reducing the heterogeneous cells in the TME and increasing the infiltration of the CD8^+^ T cells [174].

Because NPs cannot easy penetrate microvascular wall, the therapeutic effects of drugs are determined mainly by targeting and permeability. Even with both abilities, the infiltration and therapeutic effects of drugs cannot meet the requirements. Due to the strong electrostatic adsorption between the cell-penetrating peptides (cyclic peptide CRGDK/RGPD/EC) and tumor tissue, peptides together with peptide-targeted ligands can be used to assist ginsenoside penetration into a tumor to solve the problem of targeting and penetration in tumor therapy [175–177].

#### Attenuation of side-effects

Considering of the application of nanomedicines, the toxicities of NPs cannot be ignored [178–180]. The combination of NPs and ginsenosides can reduce the toxicity of nanomaterials and the side-effects of chemotherapy drugs, as well as the biodegradability of nanomaterials to achieve the efficacy of toxicity reduction. For example, the PEGylated delivery system of ginsenoside Rg3 can significantly reduce the toxicity and side-effects of nanoparticles on mice [88]. When compared with heavy-metal nanomaterials, CDs exhibited better biocompatibility and low toxicity. GO-Rh2 showed higher antitumor activity and the lowest toxicity to the coagulation system and heart tissue for the functionalization of positively charged amino acids Lys and Arg [116, 148].

In addition, Rg3 micelles can reduce the cardiotoxicity of doxorubicin [181]. The liposome G-PTX combined with ginsenoside and PTX displayed high efficiency and low toxicity because its drug content in the spleen, liver and muscles was significantly lower than was that of the traditional PTX liposome, indicating that ginsenosides can decrease the side-effects of chemotherapy drugs [27].

Biodegradability refers to the rate of biodegradation controlled by the composition and structure design of drug carriers. After the drugs with vectors are directed into the target cells, the surface carriers are biodegraded, then the drugs in the core are released to avoid releasing them into the other tissues. The materials are continuously excreted from the body through the functions of dissolution, enzymatic hydrolysis and cell phagocytosis [182]. Degradable PLA, PLGA, and PPF, have been utilized to encapsulate Rg3, 6′-O-Acetylginsenoside Rb1, and Rg1 polymeric NPs, respectively [102, 104, 123].

#### Ligand-modified drug delivery targeting

Ligand-mediated targeted DDSs can provide improvements with a variety of characteristics, including penetration across physiological barriers and into target sites and specific target cell localization [183–185]. The EPR effect, as well as recognition between ligands and receptors, has been utilized with target tumor cells to increase the curative effect. Some researchers have found that Rg3 polymeric NPs were explored for targeting cancer cells owing to their pH, and EPR effect, and longer circulation time in the blood. An intelligent mixed-micelles design with PC/DP and CK modified with amphiphilic block copolymer PEG showed sustained release and tumor cell passive targeting effects, along with good biodegradability and biocompatibility [106, 107].

To target the receptors expressed on the surface of most tumor cells, different receptors can be utilized to explore the corresponding ligands, such as molecules, antibodies, nucleic acids, and other receptor ligands (peptides, carbohydrates) [186], which can enhance NPs’ targeting function. Among them, FA is a targeting moiety in anticancer drug delivery, partly due to the high expression of folate receptors in tumor cells [128]. BSA-NPs modified with ginsenoside Rg5 and folate have been used for targeted tumor treatment. EPR effect and receptor-mediated targeting ultimately led to MCF-7 cell apoptosis [118]. In addition, peptide-A54-specific binding to liver cancer and peptide-tLyp-1-specific binding to neuropilin-1 receptor on the surface of lung cancer cells have been utilized to fabricate anti-tumor effect of compound K micelles and liposomes with pH-responsive and sustained-release properties under acidic conditions [25, 101, 109].

Other common ligands and strategies can meet the requirements with a strong targeting and penetration ability. Hyaluronic acid-modified NPs can interact with CD44 and receptors for hyaluronate mediated motility, which are overexpressed on the surface of a variety of tumor cells, while it also can be degraded by hyaluronidase around the tumor. In addition, aptamers are nucleic acids with many advantages, such as better stability and tissue permeability, high reproducibility, low cost and immunogenicity. Molecular imprinting technology can be used to design strategies for targets that are difficult to recognize [187]. For some tumor tissues, NPs’ tumor targeting specificity and selectivity can be improved by modifying the corresponding ligands. In the targeted delivery system, the recognition ability and the utilization rate of the system are limited due to the uniform distribution of the targeted part or low loading density of the targeted molecules.

In addition, the heterogeneity of tumor cells or tumor cells with low receptor expression can limit the application of targeted delivery. Therefore, strategies can be designed to increase the density and recognition ability of ligands under the stimulation of TME. For example, uniformly dispersed ligands converted into clusters in the acidic tumor microenvironment in the responding to the acidic DDS, can provide great improvements in DDS efficacy [188, 189].

#### Stimuli-responsive delivery

To precisely control drug release, the TME characteristics, including acidic pH, high glutathione content, hypoxia, and overexpressed enzymes, have been shown to be beneficial for developing a series of stimuli-responsive drugs for tumor therapy [190–192]. These NPs are usually sensitive to tiny changes related to tumor cells and TME (e.g., pH, redox state, enzymes) to release drugs, which also can be activated by external stimuli (e.g., light, heat, magnetic field, ultrasound) [193]. According to TME characteristics, conditionally sensitive NPs can achieve controlled drug release, only releasing drugs at tumor sites and stabilizing themselves under certain physiological conditions. pH-sensitive NPs (Rg3-loaded mPEG-b-P (Glu-co-Phe)) achieved CRC tumor targeting and Ph-stimulated drug release, as well as a longer circulation time in the blood, resulting in an increased tumor therapy and decreased side effects to other tissues or organs [88].

In addition, targeting NPs and microenvironment-sensitive NPs can reduce the toxicity and side-effects of ginsenoside nanomedicines. Hypoxia around the tumor tissue is caused by the lack of blood flowing through that tissue resulting from the exuberant metabolism [194]. Hypoxia-sensitive NPs can be designed to know the difference between the hypoxia environment of tumor tissue and the normoxic environment of healthy tissue. For example, although ~ 150-nm NPs exhibited good serum stability and passive tumor targeting, their weak tissue permeability can limit drug efficacy. In contrast, reducing particle size can increase tissue permeability while also reducing the stability of NPs in the blood circulation. Therefore, Designing NPs to achieve superior serum stability and permeability simultaneously is necessary.

A strategy based on the dissociation of tumor site NPs is worth pondering, which can not only increase the stability of NPs in the blood but also can enhance the tissue permeability [195]. Importantly, a human serum albumin anticancer drug modified by a hypoxia-sensitive azobenzene group has been fabricated, with a particle size of 100–150 nm. After reaching the tumor site, the azobenzene NPs groups disintegrated the NPs to < 10 nm, enhancing the role of tumor permeability and the curative effect.

#### Codelivery strategy

Combination drug therapy has synergistic therapeutic effects as compared with single chemotherapy drugs. Although it is common for multiple drugs to be applied for various diseases in the clinic, differences in physicochemical properties and pharmacokinetics may cause the drugs to fail to reach the same site at the same time, thus not achieving the optimum synergy. Therefore, it is necessary to determine the optimal ratio of drugs, which is complicated to investigate in vivo. Drug codelivery strategy provides a new possibility for the treatment of drug resistance in tumor cells.

Currently, codelivery systems including liposomes, micelles, and inorganic NPs with same pharmacokinetics have overcome multiple drug resistance, providing a new possibility for the treatment of this resistance in tumor cells. Synergistic anticancer effects produced by codelivery are of significance for DDSs..

Liposomes embedded with ginsenoside and other drugs such as curcumin, PTX, and betulinic acid can significantly inhibit tumor growth [19]. Liposomes containing parthenolide, betulinic acid, honokiol and ginsenoside Rh2 have been found to be safer than cisplatin for the treatment of tumors [98]. In addition, it has been reported that ginsenosides, as chemotherapy adjuvant and membrane stabilizer combined with PTX, could inhibit the proliferation of gastric cancer cells and reduce side-effects, thus further improving the therapeutic effect for gastric cancer. The silanol hydroxyl group on the surface and adjustable pore size of MS are beneficial to the combination of ginsenoside CK and Rh2, which exerted excellent biocompatibility with normal hacaT skin cells and anticancer effect on HepG2, A549 and HT-29 colon cancer cells [111]. Furthermore, combination therapies have displayed potential applications for reducing side-effects and metastasis and for overcoming drug resistance. Multicomponent MEs consisting of etoposide, coix seed oil and ginsenoside Rh2 have been utilized to regulate a variety of signaling pathways to overcome drug resistance by inhibiting P-gp. Furthermore, Rh2 can relieve the inflammatory response and immune suppression caused by etoposide [122]. Thus, combination therapies of ginsenosides to overcome drug resistance and metastasis require further research.

#### Photodynamic and photothermal therapy enhancement

Much attention has been focused on PDT and PTT because of their advantages of being a noninvasive, precise treatment with strong specificity and high tumor destruction efficiency [196–198]. After laser irradiation, AuNPs rapidly converted light into heat through the surface plasmon resonance effect to subject cancer cells to a high heat of 41 °C-47°C, which reduced the vitality of these cells and induced apoptosis by PTT. In PDT, also a traditional cancer treatment for AuNPs, the light of a specific wavelength is activated by photosensitizer to produce phototoxic substances in the presence of molecular oxygen, thus destroying cancer cells.

Interestingly, the surface plasmon resonance effect of GNPs in the near-infrared region can enhance the production of singlet oxygen and ROS, which can improve the therapeutic tumor effect. DCY51T-AuCKNp with CK as an effective photothermal agent has been shown to exhibit synergistic chemotherapy effects to the human lung adenocarcinoma cell line A549 and colorectal adenocarcinoma cell line HT29 [110].

In addition, the application of GO and a metal organic framework has attracted more attention; these can be used as drug delivery carriers for chemotherapy, PTT, and PDT [199–201]. Therefore, using a PPT or PDT treatment strategy for ginsenosides combined with various carriers can produce the expected effects for cancer treatment [202].

#### Disease diagnosis

One advantage of the combination of imaging and a therapy platform is that it can monitor the treatment process in real time, adjusting the treatment scheme to reduce toxicity and side-effects, ultimately achieving accurate tumor treatment [203–207]. Biomedical imaging including optical imaging [208–210], acoustic imaging [211, 212], magnetic resonance imaging (MRI) [213, 214], computed tomography (CT) and positron emission tomography (PET), is the main method for tumor diagnosis, with the aid of nanomaterials for the unique optical, electrical, and magnetic signals. Because some lesions cannot be reliably and accurately diagnosed by a single imaging method, multimodal imaging can be constructed to provide more detailed information about a tumor’s location, size, and character, thus providing support for developing a precise treatment [215].

For example, fluorescent signal probes including NIR fluorescent dye ICG [216], up-conversion nanomaterials NaYF4: Yb/Er/Tm [217], GNP clusters (AuNCs) with a particle size of < 2 nm [218], and quantum dots recently have been designed for cancer imaging and therapy due to their biocompatibility and low toxicity [219]. In addition, NPs with iron, manganese, or gadolinium ions or iron oxide NPs for MRI imaging have been applied clinically [220]. Furthermore, acoustic waves from the contrast agents for photoacoustic imaging have been shown to be much safer than those of CT or PET [221]. More complex multimodal imaging for diagnosis and designing a treatment platform are needed. Therefore, the development of ginsenosides based on an integrated imaging and therapy delivery system must be explored further.

### Multifunction as nanocarriers

**Fig. 14:**
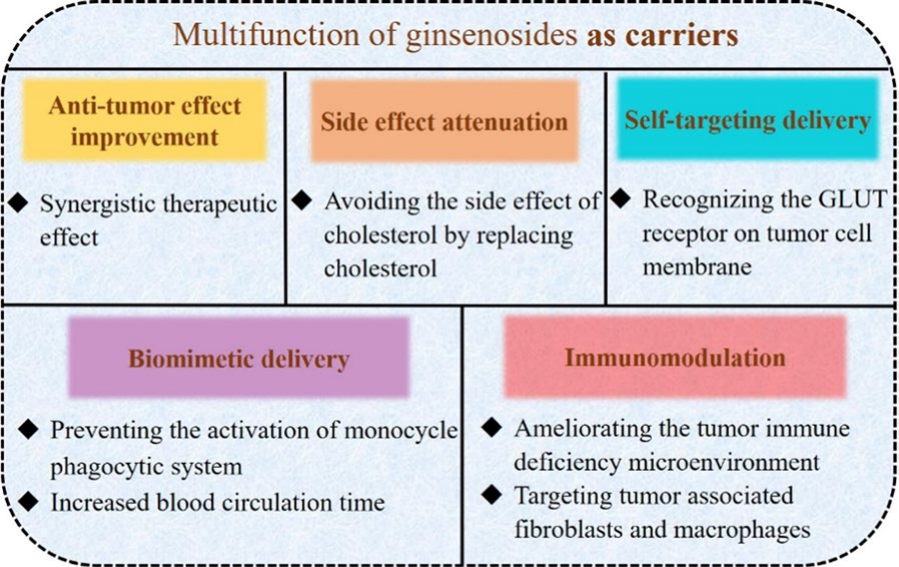
Multifunction of ginsenosides as carriers

Ginsenosides, as nanocarriers, have also shown multiple pharmacological functions, which are shown in Fig. 14 including antitumor effect improvement, side-effect attenuation, self-targeting delivery, biomimetic delivery, and immunomodulation.

#### Antitumor effect improvement and attenuation of side-effects

Most drug delivery carriers’ act only as excipients with no direct effects on the main drugs; however, short- or long-term toxicity may appear with their metabolites [108]. Ginsenosides have a potential application for being both adjuvant drugs and excipients simultaneously.

liposomes have been utilized to replace cholesterol for stabilizing the phospholipid bilayer. The liposome preparation formulations were simple without adding PEG or targeting ligand, which greatly simplified the technological production. Liposomes exerted a significant synergistic anticancer pharmacological activity with the chemotherapeutic drug PXT by targeting the GLUT on the surface of tumors [25–27].

#### Self-targeting delivery

Rh2, Rg3, and Rg5 ginsenoside liposomes can be accumulated in tumors for recognizing the GLUT receptor on the tumor cell membrane with stronger toxicities and targeting abilities. The curative effects of Rg5-PTX-liposomes have been confirmed with HGC-27, A549, and MCF-7 subcutaneous tumor models [27]. In addition, PTX-Rh2-lipo achieved excellent tumor targeting and anti-tumor activity of mouse breast cancer [25]. Rg3-PTX-liposomes inhibited tumor proliferation by activating the C6 glioma immune microenvironment via inducing the transformation of M2 TAMs into M1 in the TME [28].

#### Biomimetic delivery

The adsorption of opsonin proteins such as Ig and other complex proteins can enable NPs to be recognized and cleared easily by the mononuclear phagocytes. It has been demonstrated that the inhibited uptake of phagocytes by reducing the adsorption of these proteins can enhance the stealth effect of NPs [222].

PEGylated NPs can form steric hindrance on their surface through their hydrophilicity and spatial repulsion, thus decreasing protein adsorption and avoiding being removed by the monocyte phagocytic system [223]. Nevertheless, the increased blood circulation cycle of ginsenoside-embedded liposomes is achieved because Rh2, Rg3 and Rg5 can prevent the activation of the monocyte phagocytic system. It has been verified that the amount of Rh2 liposomes coated by protein corona was significantly lower than that of traditional liposomes [25]. The long circulating liposome fabricated with ginsenosides can reduce the adsorption of opsonins on their surface, thus exhibiting the stealth effect of ginsenoside and increasing the adsorption of apolipoprotein E, which can retard the absorption of macrophages to liposomes.

#### Immunomodulation

TME gradually has become the target of tumor treatment due to its important role in tumor development, diffusion, metastasis, and drug resistance. As compared to targeted tumor cells, one benefit of targeting nontumor cells in TME is that they are genetically more stable and, therefore, are less likely to form drug resistance. However, the difficulty of targeting nontumor cells to obtain good therapeutic effects involves reducing the toxicity to normal cells. Interfering with the TME of primary tumor cells or the premetastatic microenvironment of tumor cells can be effective for the treatment of malignant tumors prone to metastasis [224, 225].

The immunotherapeutic activities of ginsenoside liposomes have been reflected in improving the TME structure to enhance the drug permeability by reducing tumor vascular density. Some researchers have focused on transporting drugs to blood vessels with Rh2, Rh3 or Rg5 liposomes, which is important for tumor growth and metastasis [25, 26, 28]. Targeted stromal cells including cancer-associated fibroblasts and TAMs also have been utilized for cancer treatment [174]. Ginsenosides liposomes changed the immune-deficiency TME mainly through reducing the heterogeneous cells in the TME and enhancing the immune function by increasing the infiltration of CD8^+^ T cells. As a targeted stromal cells, TAMs easily differentiate into M2 phenotypes, which often have been associated with tumor metastasis and poor outcomes. Ginsenoside liposomes have played a role in reconstructing the TME by transforming TAM2 into TAM1 to promote the role of T cells by inhibiting the activities of signal transducers and transcription activators [25].

### Multifunction as both drug and nanocarrier

Ginsenosides as a drug are commonly encapsulated into the core of NPs or modified on their surface. Ginsenoside Rg3, Rg5, and Rh2 have acted as carriers of nano lipid structures, substituting cholesterol for the similar structure. Therefore, ginsenoside DDSs not only can exhibit their multifunctionality as a drug but also can display their merits as carriers.

It has been verified that ginsenoside nanomedicines can increase anticancer efficacy and can exhibit synergistic anticancer effects with other chemotherapy drugs while decreasing the side-effects caused by these drugs, NPs, or cholesterol. In addition, the ligand-targeting ability of ginsenoside DDS for specific sites or their self-targeting ability for recognizing the GLUT receptor on the tumor cell membrane enable ginsenosides to be potential delivery systems. Furthermore, ginsenosides themselves are endowed with the delivery system’s stealth effect for the inhibition of the uptake of phagocytes and immune function by increasing the infiltration of CD8^+^ T cells and reconstructing the TME, strengthening tumor treatment.

## Clinical application and translation

Ginsenoside nanomedicines have shown great application value and development prospects. Certain tumor therapeutic nanomedicines are in clinical trials, such as a G-PTX liposome loaded with ginsenoside and PTX, and have exhibited advantages for drug loading, tumor targeting, stability, and biological safety [27]. However, much remains to be explored before these therapies are truly mature. Several challenges must be solved before applying ginsenoside DDSs in clinical practice [226–228], including the synthesis and large-scale production of controllable and repeatable nanodrugs, the development of a safety evaluation, and the undertaking of clinical research.

### Nanodrug synthesis and large-scale production

Repeatable synthesis methods and controllable quality are prerequisites for drug clinical transformation. Some multifunctional nanodrugs (e.g., layer-by-layer, self-assembly NPs) involve multiple or complex synthesis steps. Therefore, it is difficult to synthesize nanodrugs with the same qualities quickly, accurately and repeatedly. Microfluidic technology might help solve the problem of inconsistent effects from laboratory research to clinical experiments given its high-speed self-assembly, narrow size distribution, and good repeatability [229].

In addition, monodisperse NPs with highly controllable size, shape, chemical composition, surface properties, and drug loading capacity can be synthesized using a nonwetting template complex method [230]. To carry out a comprehensive quality control for nanodrugs and carrier materials, more research on their formulation technology and stability are necessary. Furthermore, corresponding quality control methods must be established, optimized, and verified. The research and development of a GMP-compliant preparation process and equipment for the large-scale production of nanodrugs will be required.

### Safety evaluation and clinical research

As compared with traditional small-molecule or biological macromolecular drugs, ginsenoside nanodrugs are different in their variety and physical and chemical properties. Hence, to achieve a clinical transformation, we must focus on evaluating the biocompatibility and safety of NPs in vivo. Safety assessment first requires the completion of high-throughput cytotoxicity testing, including for oxidative stress, surface membrane, and mitochondrial damage, lysosomes, autophagy, and inflammatory corpuscles. Animal models with different species are needed to study the pharmacokinetics, biodistribution, efficacy, and safety of nanomedicines [226]. It will be necessary to carry out preclinical studies on ADME, toxicokinetics, acute toxicity, immunology, and drug-specific toxicity of drugs in vivo, as well as to design standard operating procedures for safety evaluation method.

Certain correlations and differences exist between the toxicological results of experimental animals and the adverse reactions of the human body. Although animal models are commonly used to evaluate safety, the safety responses of animals and humans are different and can be affected by many factors, such as the differences of cytochrome P450 and enzymes. There also are differences among the animal models themselves. For example, the rat model is more sensitive than is the mouse model for predicting the GI toxicity of chemotherapy and targeted therapy. Some models, such as the genetically engineered and patient-derived xenotransplantation mouse model, can accurately simulate the heterogeneity of human tumors [231, 232].

Another challenge is clinical trials. After the completion of a preclinical safety assessment, it will be necessary to evaluate and manage patient side-effects and adverse events to evaluate toxicity in the human body, so as to facilitate the successful clinical transformation of the drugs.

## Conclusion and prospects

The ideal DDS maintains a drug concentration in a certain range or specifically delivers a drug to target organs and tissues after a single administration while minimizing the drug’s concentration in other regions and reducing the immune response in vivo. Ginsenosides have been an important component of cancer drugs for a long time. The research on and development of ginsenoside DDSs have provided a certain prospect for tumor treatment, but the antitumor mechanisms of some nanodrugs require further research. Only with a reasonable research strategy, comprehensive preclinical evaluation and strict clinical trials can more antitumor drugs be developed.

The antitumor effects of a ginsenoside DDS for lung, colon, and liver cancer cells, as well as the inhibitory effects on tumor xenografts in mice, have been investigated. Although the nano-delivery systems can increase drug concentration in the blood, only a few studies have explored in depth the distribution of ginsenosides or their metabolites in tumor tissues or organs. The research on ginsenoside DDSs is still in the preclinical stage, and no DDS has been launched successfully. Although NPs have achieved progress, the clinical transformation of nanodrugs for cancer treatment remains a focus.

To simulate the real clinical environment as much as possible, it is necessary to further explore the mechanism and pharmacokinetics of a ginsenoside DDS in vivo to inhibit cancer metastasis and to combine chemical sensitizers to eliminate the resistance of the body to ginsenosides. Furthermore, the therapeutic effects can be maintained by a combination of ginsenosides and classical chemotherapy drugs, the dosage of which can be reduced. Although still in the early stages of research and development, a multifunctional DDS for diagnosis, treatment, and prognosis will require evaluating its efficacy in vivo*,* which is worthwhile research. The development and improvement of new dosage forms show a great potential in cancer treatment.

## Data Availability

Not applicable.
